# Clonal Progression during the T Cell-Dependent B Cell Antibody Response Depends on the Immunoglobulin D_H_ Gene Segment Repertoire

**DOI:** 10.3389/fimmu.2014.00385

**Published:** 2014-08-11

**Authors:** Ahmad Trad, Radu Iulian Tanasa, Hans Lange, Michael Zemlin, Harry W. Schroeder, Hilmar Lemke

**Affiliations:** ^1^Biochemical Institute of the Medical Faculty of the Christian-Albrechts-University, Kiel, Germany; ^2^Philipps-University of Marburg, Marburg, Germany; ^3^Department of Medicine, The University of Alabama at Birmingham, Birmingham, USA

**Keywords:** rodent, B cells, antibodies, class switch recombination, repertoire development

## Abstract

The diversity of the third complementarity determining region of the IgH chain is constrained by natural selection of immunoglobulin diversity (D_H_) sequence. To test the functional significance of this constraint in the context of thymus-dependent (TD) immune responses, we immunized BALB/c mice with WT or altered D_H_ sequence with 2-phenyloxazolone-coupled chicken serum albumin (phOx-CSA). We chose this antigen because studies of the humoral immune response to the hapten phOx were instrumental in the development of the current theoretical framework on which our understanding of the forces driving TD responses is based. To allow direct comparison, we used the classic approach of generating monoclonal Ab (mAb) from various stages of the immune response to phOx to assess the effect of changing the sequence of the D_H_ on clonal expansion, class switching, and affinity maturation, which are hallmarks of TD responses. Compared to WT, TD-induced humoral IgM as well as IgG antibody production in the D-altered ΔD-DμFS and ΔD-iD strains were significantly reduced. An increased prevalence of IgM-producing hybridomas from late primary, secondary, and tertiary memory responses suggested either impaired class switch recombination (CSR) or impaired clonal expansion of class switched B cells with phOx reactivity. Neither of the D-altered strains demonstrated the restriction in the V_H_/V_L_ repertoire, the elimination of V_H_1 family-encoded antibodies, the focusing of the distribution of CDR-H3 lengths, or the selection for the normally dominant Ox1 clonotype, which all are hallmarks of the anti-phOx response in WT mice. These changes in clonal selection and expansion, as well as CSR indicate that the genetic constitution of the D_H_ locus, which has been selected by evolution, can strongly influence the functional outcome of a TD humoral response.

## Introduction

In immunoglobulins, juxtaposition of the three complementary determining regions (CDRs) of the L chain and the three of the H chain creates the site at which antigen binds (1, 2). While CDRs 1 and 2 are entirely of germline origin and CDR-L3 is largely so, CDR-H3 is the direct product of VDJ rearrangement and N nucleotide addition (3). This makes CDR-H3 the focus for pre-immune Ig diversity. In combination, this diversity and its physical location at the center of the antigen binding site tends to endow CDR-H3 with the ability to define the antigen binding specificity and affinity of the antibody.

Analyses of anti-hapten immune responses have been crucial for the dissection of the roles played by T cells in initiating and regulating humoral immune maturation. Immune maturation in the classic humoral immune response of BALB/c mice to the hapten 2-phenyloxazolone (phOx) (4) focuses on the clonal expansion and somatic hypermutation of Ig bearing the dominant Ox1 Id (Id_Ox1_). While this Id is marked by the use of a combination of V_H_Ox1 and V_κ_Ox1 variable genes, the presence of a short DRG peptide sequence in CDR-H3 is typically determinative (4, 5).

To test the role of natural selection of D gene segment sequence on humoral immune function, we previously created a panel of BALB/c-derived D-altered mutant mouse strains (6–8). ΔD-DμFS and ΔD-iD B cells produce two alternative, polyclonal Ig repertoires with a normal and intact set of V_H_, J_H_, and C_H_ exons that are fully capable of undergoing somatic hypermutation and class switching (6, 8). The only change that has been made is the simplification of D_H_ locus to contain only one D of alternative sequence. After VDJ rearrangement, even the loxP sites are deleted, leaving only the imprint of the three to seven amino acids encoded by the D_H_. The CDR-H3s that contain identifiable D_H_ sequence create an antigen binding site repertoire that differs greatly in the pattern of amino acid use from WT. However, CDR-H3 sequences that lack identifiable D_H_ sequence and are created by V, J, and N sequence alone appear indistinguishable from similar sequences created in wild-type (WT) mice (Figures S1 and S2 in Supplementary Material).

The DRG peptide sequence characteristic of the dominant Ox1 Id is an example of a CDR-H3 that can be easily created either with or without D gene segment sequence. The nine nucleotides used to encode DRG can include three to five nucleotides from 5 of the 13 D_H_ gene segments. However, the DRG sequence can also be created by simply introducing five N nucleotides between V_H_Ox1 and J_H_3. Our panel of D-altered mice thus provided us with the means to test the extent to which loss of the naturally selected D-dependent CDR-H3 repertoire would influence the development of a classic T dependent response to a defined hapten even when the loss of D sequence could be easily mitigated by N addition alone.

To allow direct comparisons to previous studies, we used the classic approach of generating monoclonal Ab (mAb) from various stages of the immune response to phOx. We found that changing conserved elements of the sequence of the diversity gene segment locus led to the failure to select for the use of V_H_Ox1/V_κ_Ox1 gene combination, the failure to yield the normal focusing of CDR-H3 sequence, and thus the loss of Id_Ox1_ dominance. Further, we observed an enhanced and persistent production of hybridomas secreting low affinity IgM indicating a profound failure to develop a fully mature, class switched IgG response. Together, these findings suggest that TD B cell responses can be heavily influenced by the effects of natural selection of D_H_ content on CDR-H3 repertoire diversity.

## Materials and Methods

### Animals

Wild-type female BALB/c (H-2^d^) mice (Harlan-Winkelmann; Borchen, Germany) and BALB/c D-altered homozygous ΔD-DμFS (7) and ΔD-iD (6) mice were reared under clean conventional conditions the University of Kiel animal house. Immunizations and experimental procedures were approved by the “*Ministry of Agriculture, Environment and Rural Areas*” of the local government of Schleswig-Holstein, permission no. V 312-72241.121-3 (35-3/06).

### Antigens, immunizations, antibody titrations, and production of monoclonal antibodies

BALB/c WT and D-altered ΔD-DμFS and ΔD-iD mice were immunized with the TD hapten–protein complex phOx-coupled chicken serum albumin (phOx-CSA) (molar ratio ~11) (9, 10). At 3–4 months of age, animals of all three strains received 80 μg of phOx-CSA adsorbed to Al(OH)_3_ as a primary intraperitoneal immunization. Venous blood was serially obtained by tail vein phlebotomy and serum antibody concentrations were determined after primary or secondary immunization. PhOx-binding IgM and IgG titers were determined with ELISA using class-specific secondary antisera. Spleen cells of phOx-CSA-immunized WT or D-altered mice were fused with the non-secretor Ag8.653 myeloma cells by means of the conventional PEG-mediated hybridization technique. Fusions were performed 7 or 14 days after primary immunization and 3 days after each secondary (memory) immunization. Resulting mAb reactive with phOx-BSA but not with BSA alone were selected for further study.

### Determination of relative affinities of anti-phOx antibodies

The relative affinities of mAb antibodies to phOx were determined with a hapten-inhibition test as previously described (9). These affinities were assessed in comparison to that of two prototypic Ox1-idiotypic mAb: H11.5 (μ,κ) for IgM and NQ2/16.2 (γ,κ) for IgG Ab. Briefly, the binding of comparable amounts of anti-phOx mAb to surface-bound phOx-BSA was inhibited with graded concentrations of soluble phOx-caproic acid. Concentration values giving 50% inhibition were taken as relative affinity measures. An affinity factor was generated as the quotient of the relative affinity of H11.5 (μ,κ) for IgM and NQ2/16.2 (γ,κ) for IgG mAb divided by that of a particular mAb. MAb with higher affinity than the controls exhibited an affinity factor greater than 1, and those with a lower affinity exhibited an affinity factor of less than 1. Independent confirmation of these measures of affinity was obtained by fluorescence quenching (10).

### Sequencing of antibody V region genes

Sequence analysis of the variable regions of mAb was performed as previously described (11) with some modifications. Briefly, total RNA of anti-phOx Ab secreting hybrid cell lines was isolated with TRIZOL^®^ (GIBCO-BRL, Eggenstein, Germany) and transcribed into cDNA with SuperScript^TM^ II RNAse H reverse transcriptase (GIBCO-BRL, Eggenstein, Germany) using pd(N)6 random and pd(T)_12–18_ primers. The V_H_ and V_L_ mRNA sequences were first amplified by PCR using 10 primer sets for each of the V_H_-regions, and 7 primer sets for each of the V_L_-regions and two forward primers specific for the 3′-end for the first domain of the V_H_ and V_L_ constant regions, respectively. A second semi-nested amplification at 3′-end was then performed with relevant primers coupled to M13 oligonucleotides. This amplimer product was then used for sequencing (MWG Biotech; Ebersberg, Germany). V region sequences were analyzed with the integrative database VBASE2 (http://www.vbase2.org/).

### Statistical analysis

One-way analysis of variance (ANOVA) followed by Dunnet’s multiple comparison post test, using Graphpad Prism for Windows (version 4.02, GraphPad Software Inc., San Diego, USA) was used for statistical comparisons of individual IgM and IgG end-point titers.

### Accession numbers

GenBank accession numbers for all antibodies are indicated in the supplementary table legends of each group of antibodies.

## Results

### Humoral immune response and analysis of 2-phenyl-5-oxazolone-specific monoclonal antibodies

The humoral Ab response to phOx in BALB/c WT mice was compared to that of homozygous D-altered ΔD-DμFS and ΔD-iD mutant mice. The D_H_ locus in these strains was first simplified by deleting 12 of the 13 D_H_ gene segments by means of cre/loxP gene-targeting (12); and then modifying the sequence of the remaining D_H_. In the ΔD-DμFS strain, the WT sequence of the DFL16.1 D_H_ segment was modified by introducing two frame-shift mutations that flipped the normal preference for reading frame 1 (RF1), which encodes neutral tyrosine and glycine, to RF2, which encodes hydrophobic valine. In the ΔD-iD strain, the center of the DFL16.1 segment was replaced with the DSP2.2 D_H_ gene segment in inverted form. This leads to CDR-H3 enriched for charged arginine, asparagine, and histidine in place of tyrosine and glycine (6, 8).

**Figure 1:**
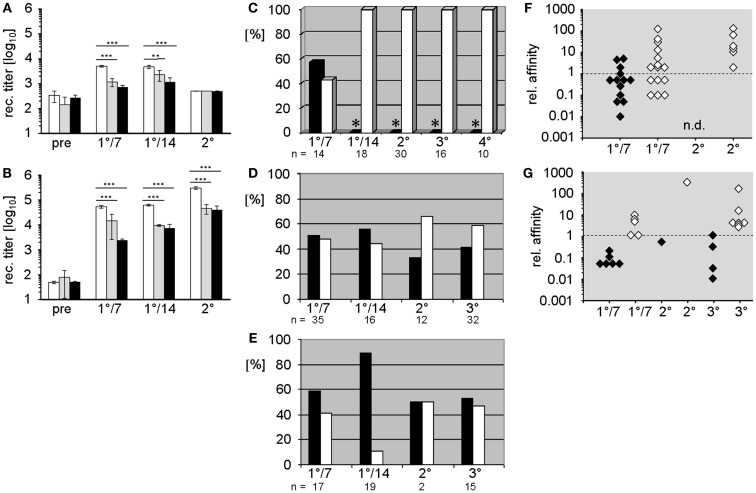
**Comparison of humoral Ab responses to the hapten 2-phenyloxazolone in BALB/c wild-type and D-altered ΔD-DμFS and ΔD-iD mutant mice**. **(A,B)** IgM **(A)** and IgG **(B)** anti-phOx Ab titers of primary and secondary thymus-dependent immune responses after immunization with phOx-CSA in BALB/c wild-type mice (white bars), ΔD-DμFS mice (gray bars), and ΔD-iD mice (black bars). The bars show the mean + SD of five mice per group. Pre – pre-immune sera; 1°/7 – primary day 7 and 1°/14 – primary day 14 responses. Secondary immunization followed 14 weeks after primary and Ab titers were determined after two more weeks. Significance is indicated at **p < 0.01; ***p < 0.001. **(C–E)** Monoclonal anti-phOx mAb were prepared from **(C)** BALB/c wild-type, **(D)** ΔD-DμFS, and **(E)** ΔD-iD mice on day 7 (1°/7) and day 14 (1°/14) after primary immunization with phOx-CSA and 3 days after secondary (2°), tertiary (3°), and from wild-type mice also after quaternary (4°) immunizations. For ΔD-DμFS and ΔD-iD mice, percentages of IgM and IgG mAb are indicated as black and white bars, respectively. However, because extremely low numbers of IgM mAb have been isolated from the late primary and memory responses of BALB/c wild-type mice (*), only IgG mAb have been produced. **(F,G)** The relative affinities of IgM (black diamonds) and IgG (white diamonds) anti-phOx mAb from different stages of the immune response of **(F)** ΔD-DμFS and **(G)** ΔD-iD mutant mice are compared either to the IgM Id_Ox1_ mAb H11.5 (μ,κ) or to the IgG Id_Ox1_-prototypic mAb NQ2/16.2 (γ,κ). Affinity factors of >l indicate higher while affinity factors of <l indicate lower affinities than H11.5 and NQ2/16.2, respectively.

In both mutant strains, primary immunization with the TD Ag phOx-CSA-induced significantly lower IgM and IgG anti-phOx Ab titers than WT (Figures 1A,B). Following secondary (memory) immunization, the IgG response remained suppressed while IgM production was meager and indistinguishable between all three strains.

Study of the molecular events during immune maturation of the TD anti-phOx response classically focused on analysis of mAb (9, 13, 14) secreted by hybridomas produced by fusion of permanently growing myeloma cells with immune splenocytes obtained after sequential primary and memory immunization (9, 13, 14). In order to directly compare the effect of altering D_H_ locus content in the same classic context, we generated anti-phOx mAb-secreting hybridomas from immune splenocytes harvested after primary, secondary, and tertiary immunizations of D-altered ΔD-DμFS and ΔD-iD mutant mice.

The characteristic parameters of these mAb, including isotype, relative affinity, usage of V_H_/V_L_ genes and D_H_ and J_H_ gene segments, amino acid sequence, and reading frame usage in CDR-H3, and correspondence with the normally dominating Id_Ox1_ are summarized Tables S1 and S2 in Supplementary Material. A comparison of these mAb with those of previously published mAb (10) from WT mice revealed crucial differences induced by the change in D_H_ sequence content. Unlike WT mice, where the majority of hybridomas ( >80%) isolated during late primary responses and on day 3 of secondary and tertiary responses produce IgG, and thus reflect T cell aided CSR (15); only half of the hybridomas obtained from the D-altered ΔD-DμFS and ΔD-iD mice produced IgG. This included both the hybridomas generated after the late primary response as well as hybridomas harvested from splenocytes 3 days after memory immunizations (Figures 1D,E). Thus, the normal pattern of favoring production of IgG secreting B cells failed to occur in mice lacking a WT D_H_ locus, and therefore, a WT CDR-H3 repertoire. A full depiction of these antibodies is provided in Tables S1 and S2 in Supplementary Material.

To assess the quality of these mAb, we compared their relative affinities to those of prototypic IgM and IgG Id_Ox1_ mAb. Early primary IgM mAb of both mutant strains, as well as those from secondary and tertiary responses, were mostly of rather low affinity (Figures 1F,G). However, three IgM mAb from ΔD-DμFS mice exhibited higher affinities than the IgM Id_Ox1_ mAb H11.5 (Figure 1F), even though none of them included the Id_Ox1_ V gene combination of V_H_Ox1/V_κ_Ox1, which is designated V_H_171/V_κ_072 in the integrative database VBASE2.

Early primary IgG mAb generally exhibited higher affinities than the IgM mAb, with some IgG mAb displaying even higher affinities than the IgG Id_Ox1_ mAb NQ16.2. However, none of the mAb expressed the Id_Ox1_ V_H_Ox1/V_κ_Ox1 gene combination. IgG of higher affinity were found among secondary and tertiary response mAb. However, immune maturation did not progress in the steady way that characterizes the classic response of WT mice (see below).

**Figure 2:**
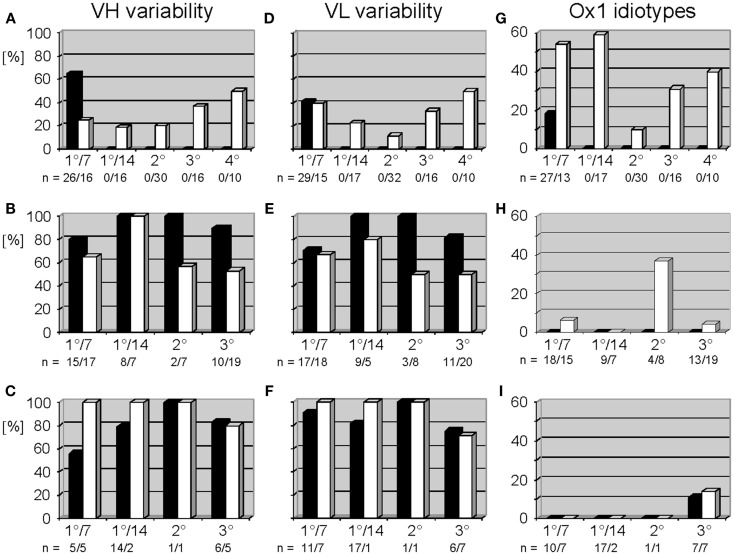
**Variable gene usage of mAb of the thymus-dependent anti-phOx immune response in BALB/c wild-type and ΔD-DμFS and ΔD-iD D_H_ mutant mice**. Mice were immunized with the TD Ag phOx-CSA and mAb were prepared on day 7 (1°/7) and day 14 (1°/14°) after primary immunization and 3 days after secondary (2°), tertiary (3°), and quaternary (4°) immunizations, respectively. The characteristic attributes and utilized variable genes of these Ab are depicted in Tables S1 and S2 in Supplementary Material. Variability is calculated as the number of genes expressed by a group of antibodies divided by the number of monoclonal antibodies of this group and multiplied by 100. In BALB/c mice, IgM mAb (black bars) were only prepared early after primary immunization; see also legend to Figure 1. Data for IgG (white bars) are taken from two previous publications (9, 10). V_H_ and V_L_ variability is indicated for anti-phOx mAb of BALB/c wild-type mice **(A,D)**, ΔD-DμFS mice **(B,E)**, and ΔD-iD mice **(C,F)**. **(G–I)** Expression of Ox1-idiotypic gene combination VH171/Vκ072 by monoclonal anti-phOx Ab from the TD response in BALB/c wild-type mice **(G)**, ΔD-DμFS mice **(H)**, and ΔD-iD mice **(I)**. Data of BALB/c wild-type mice are taken from previous publications (5, 9, 10).

In accordance with historical studies, in WT mice the anti-phOx response exhibited a drastic CSR-associated reduction in the variability of the V_H_ and V_L_ repertoires (10) (Figures 2A,D), especially in the class switched secondary response. No such reduction in V_H_ or V_L_ variability was observed in the D-altered ΔD-DμFS and ΔD-iD mice (Figures 2B,E and C,F). The classic secondary phOx response in WT mice is associated with a shift away from use of the V_H_1 family (10). However, in the D-altered mice hybridomas secreting V_H_1 family-encoded IgM and IgG mAb continued to be isolated at all phases of immune maturation (Tables S1 and S2 in Supplementary Material).

In WT mice, the TD response to phOx-CSA is dominated by V_H_171/V_κ_072-encoded Id_Ox1_ Ab (4) whose idiotypic determinant is located in CDR-H3 (5). A compilation of previous data (9, 10) shows that mAb with the gene combination V_H_171/V_κ_072 (the “genetic” Id_Ox1_) first dominate after CSR among IgG mAb (Figure 2G). This gene combination is counter-selected in secondary response mAb, but increases again in tertiary and quaternary responses. However, in both of the D-altered strains the Id_Ox1_ gene combination V_H_171/V_κ_072 only represented a minority of clones. Among ΔD-DμFS mAb, the V_H_171/V_κ_072 gene combination was not identified among the IgM hybridomas. It was detected in IgG mAb drawn from the primary response, but at a low level. A plurality of the secondary IgG response used this combination, but this was again lost in the tertiary response (Figure 2H). In ΔD-iD mice, anti-phOx antibodies with the V_H_171/V_κ_072 gene combination were not observed in either the primary or secondary responses. It was found among tertiary IgM and IgG, but again at a low level (Figure 2I).

### Analysis of CDR-H3 of monoclonal anti-2-phenyl-5-oxazolone antibodies

**Figure 3:**
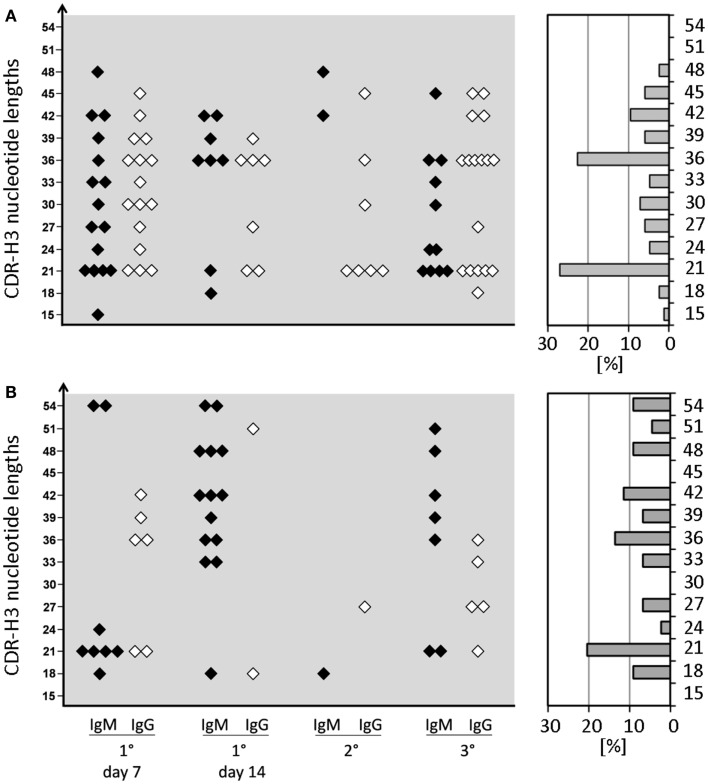
**CDR-H3 nucleotide lengths of IgM and IgG mAb of the anti-phOx immune response of D-altered ΔD-DμFS and ΔD-iD mutant mice**. Monoclonal Ab were prepared from mice that were immunized various times with the TD Ag phOx-CSA (see previous figures). The characteristic attributes of these Ab are depicted in Tables S1 and S2 in Supplementary Material. The CDR-H3 nucleotide lengths of IgM (black symbols) and IgG (white symbols) monoclonal anti-phOx Ab from different stages of the immune response of ΔD-DμFS mice are shown in **(A)** and those of ΔD-iD mice in **(B)**. The percental contribution of each nucleotide length is indicated by gray bars at the right hand side.

In WT mice, CSR and affinity maturation are strongly associated with a focusing of CDR-H3 content. For example, whereas the CDR-H3 of anti-phOx IgM hybridomas varied between 15 and 51 nucleotides, 70–100% of primary and memory IgG mAb exhibited a clearly dominating length of 21 nucleotides (10). This CSR-associated restriction of CDR-H3 lengths was not observed in D-altered ΔD-DμFS and ΔD-iD mice (Figure 3). CDR-H3 lengths with 21 and 36 nucleotides were slightly increased among ΔD-DμFS mAb (Figure 3A) and to an even lesser degree among ΔD-iD mAb (Figure 3B). Otherwise, IgM as well as IgG mAb from both strains of mice demonstrated a wide distribution of lengths.

**Figure 4:**
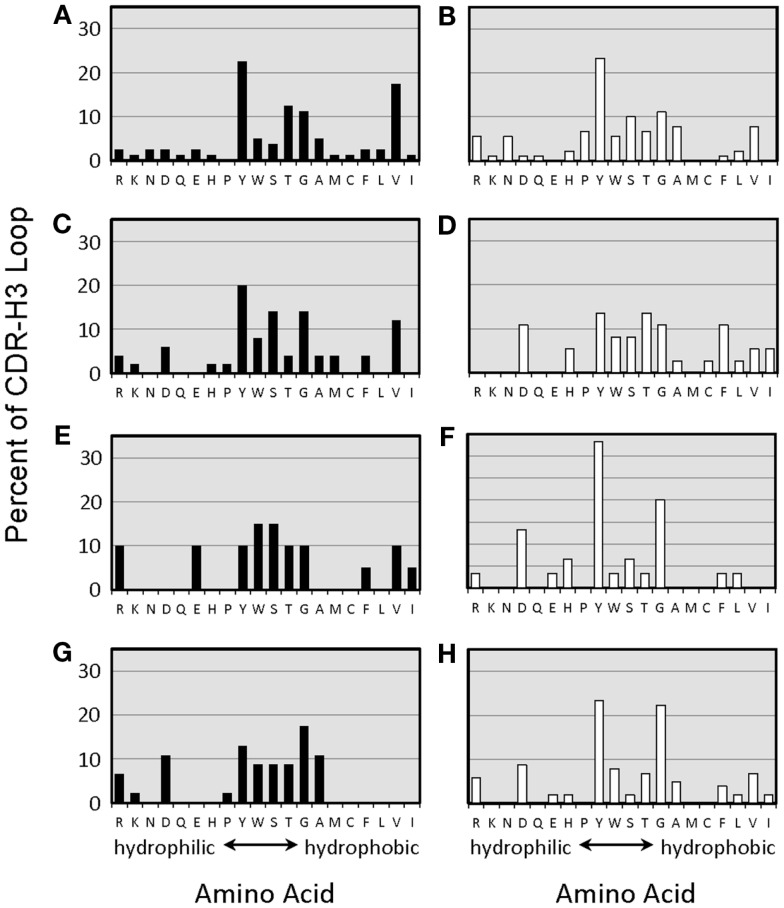
**Amino acids of CDR-H3 loops of monoclonal anti-phOx Ab of D-altered ΔD-DμFS mice**. Amino acids are indicated at the *x*-axis in the one-letter code. Hybridomas secreting anti-phOx antibodies (IgM black bars, IgG white bars) were generated after primary immunization on day 7 **(A)** IgM (*n* = 16 mAb), **(B)** IgG (*n* = 14 mAb), and on day 14 **(C)** IgM (*n* = 8 mAb), **(D)** IgG (*n* = 7 mAb), 3 days after secondary immunization **(E)** IgM (*n* = 2 mAb), **(F)** IgG (*n* = 7 mAb), 3 days after tertiary immunization, **(G)** IgM (*n* = 11 mAb), and **(H)** IgG (*n* = 17 mAb). See also Figure S3 in Supplementary Material.

**Figure 5:**
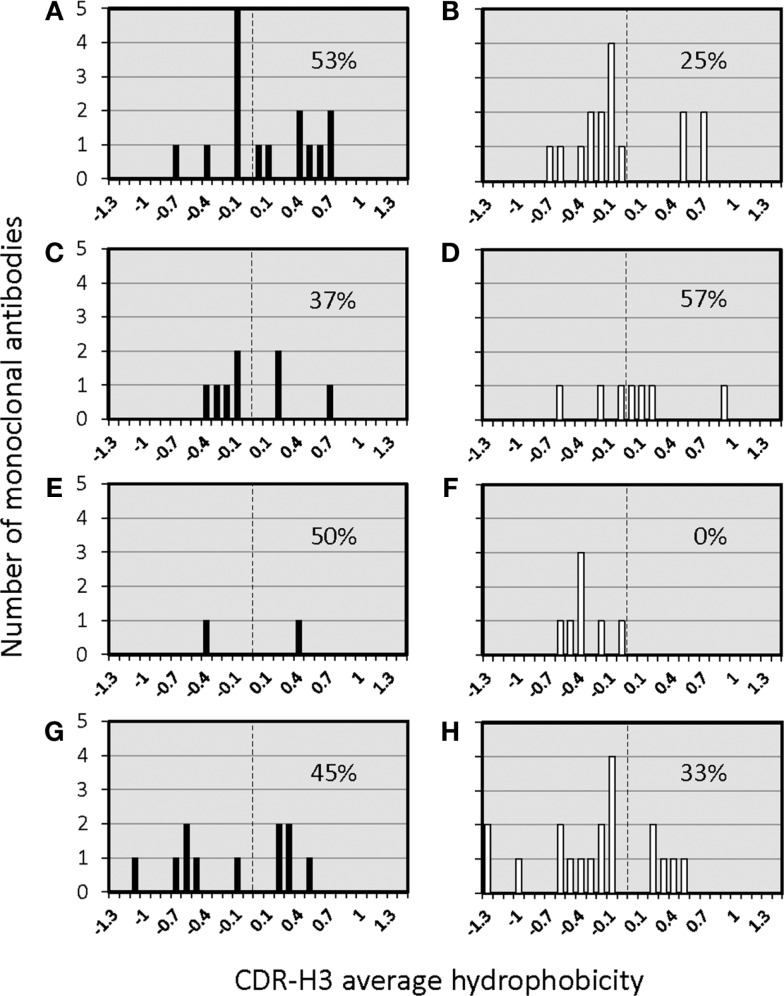
**Distribution of average hydropathicity values of CDR-H3 loops of monoclonal anti-phOx Ab of D-altered ΔD-DμFS mutant mice**. The average hydropathicity values of CDR-H3 loops were calculated with the normalized Kyte–Doolittle hydrophobicity scale (8). Anti-phOx antibody-secreting hybridomas (IgM black bars, IgG white bars) were generated after primary immunization on day 7 **(A)** IgM (*n* = 16 mAb), **(B)** IgG (*n* = 14 mAb), and on day 14 **(C)** IgM (*n* = 8 mAb), **(D)** IgG (*n* = 7 mAb), 3 days after secondary immunization **(E)** IgM (*n* = 2 mAb), **(F)** IgG (*n* = 7 mAb), 3 days after tertiary immunization, **(G)** IgM (*n* = 11 mAb), and **(H)** IgG (*n* = 17 mAb). See also Figure S3 in Supplementary Material.

The distribution of charged, neutral, and hydrophobic amino acids dictates the average hydropathicity of the CDR-H3 loop. During immune maturation among WT mice, highly hydrophobic amino acids found among IgM anti-phOx CDR-H3 are gradually supplanted by arginine, aspartic acid, glycine, and tryptophan (Figures S3A–H in Supplementary Material). In contrast, from the primary to tertiary responses in both ΔD-DμFS mice (Figure 4) as well as ΔD-iD mice (data not shown), hydrophobic amino acids were found to a similar extent in the CDR-H3 of both IgM and IgG anti-phOx antibodies. The content of hydrophobic amino acids in the CDR-H3 loops was also reflected in the corresponding hydrophobicity values. In anti-phOx mAb from WT mice, about 25% of IgM mAb exhibited CDR-H3 with positive average hydropathicity values, whereas only 6% of IgG antibodies (with the exception of secondary IgG) belonged to this category (Figures S4A–H in Supplementary Material). In contrast, IgM as well as IgG mAb produced by ΔD-DμFS B cells expressed similar proportions of CDR-H3 loops with positive average hydropathicity values (Figure 5). [The numbers of mAbs generated from the ΔD-iD mice were insufficient to allow a firm conclusion on whether average hydrophobicity was maintained or altered in this strain (data not shown).]

Since idiotypic determinants defining Id_Ox1_ antibodies are located in CDR-H3, we analyzed antibodies bearing this idiotype to assess, which D_H_ gene segments could be used to generate the typical Id_Ox1_ CDR-H3 sequence C_ARDRGAY. In solely phOx-CSA-induced mAb from WT mice (10), this sequence could be generated from the five D_H_ gene segments DSP2.2, DSP2.9, DSP2.11, DST4, and DQ52 (Table 1). In contrast, after a primary immunization with the TI-2 Ag phOx-Ficoll and a subsequent TD immunization (primary to quaternary) with phOx-CSA, CDR-H3 sequences from anti-phOx mAb were drawn from a larger set of eight D_H_ segments (DSP2.2, DSP2.3, DSP2.5, DSP2.6, DSP2.9, DSP2.10) plus two sequences from inverted DFL16.1 and DFL 16.2, respectively (9). Moreover, in half of the mAb, the D_H_ segment could not be identified due to extensive nibbling (9). This raised the issue of whether ΔD-DμFS mutant mice bearing a frameshifted DFL16.1 D_H_ segment or ΔD-iD mice with an inverted DSP2.2 gene sequence would be able to generate a typical Id_Ox1_ CDR-H3 sequence in association with the VH171 gene.

**Table 1:** **D_H_ segment usage of IGVH171-encoded monoclonal anti-2-phenyloxazolone antibodies from BALB/c wild-type mice immunized with two different immunization schemes**.

	FL16.1	FL16.2	SP2.2	SP2.3	SP2.5	SP2.6	SP2.9	SP2.10	SP2.11	ST4	Q52	Not found
**Scheme 1** (*n* = 22)	–	–	1^a^	–	–	–	6	–	6	2/3^b^	4/3	–
**Scheme 2** (*n* = 39)	–/1	–/1	2	2	1	1/1	4/2	4	–	–	–	20^c^

*Scheme 1 – usage of D_H_ segments of monoclonal antibodies prepared from mice immunized with the thymus-dependent antigen 2-phenyloxazolone-(phOx)-coupled chicken serum albumin (phOx-CSA) (3)*.

*Scheme 2 – here, monoclonal antibodies are combined, which were generated in the course of the TD response to phOx-CSA of mice previously immunized with the TI-2 antigen phOx-Ficoll (12). Both groups comprise antibodies from the early primary to hyper-immune responses*.

*^a^Numbers without or before a slash indicate the amount of antibodies using the respective D_H_ gene segment*.

*^b^Numbers behind the slash indicate usage of inverse D_H_ gene segments*.

*^c^Assignment of a D_H_ gene segment was not possible*.

C_ARDRGAY-related sequences were identified (Table 2A). The majority of these CDR-H3s lacked identifiable D_H_ sequences and instead was largely the product of N and P nucleotide addition. Among the mAbs drawn from the ΔD-DμFS mice, two contained CDR-H3 amino acids coded by the frameshifted DFL16.1 gene segment. Five additional ΔD-DμFS mAb (FS1°14/05, FS3°/08, FS3°/09, FS3°/22, FS3°/18) also exhibited CDR-H3 sequences already found in Id_Ox1_ antibodies from WT mice, but these mAbs used N and P nucleotides to recreate the sequence. Among the mAbs drawn from the ΔD-iD mice, one early primary mAb, iD1°7/06, exhibited the correct Id_Ox1_ sequence in CDR-H3. These findings made it clear that both D-altered strains were able to generate Id_Ox1_-typical or related CDR-H3 sequences in spite of the alteration in D_H_ sequence content. Thus, differences in the CDR-H3 repertoire in these D-altered strains cannot be simply explained by an inability to generate the classic Id_Ox1_ sequence.

**Table 2:** **CDR-H3 amino acid sequences of (A) V_H_171- and (B) non-V_H_171-encoded monoclonal anti-phOx antibodies from D-altered ΔD-DμFS and ΔD-iD mutant mice^a^**.

ΔD-DμFS mice					ΔD-iD mice				
mAb^b^	Is^c^	Fam^d^	V_H_^e^	CDR-H3^f^	mAb^b^	Is^c^	Fam^d^	V_H_^e^	CDR-H3^f^
**(A)**
1°7/09	μ	2	171	C_AR***LTQT***FAY	1°7/04	μ	2	171	C_AR***DRG***DY
1°7/24^g^	γ	2	171	C_AR***DPG***AY	1°7/05	μ	2	171	C_SR***DRG***DY
1°14/11	γ	2	171	C_AR***DF******G******K***D	1°7/06	μ	2	171	C_AR***DRG***AY
2°/05^g^	γ	2	171	C_AR***DSG***DY	1°7/12	γ	2	171	C_AR***DRG***DY
2°/06^g^	γ	2	171	C_AR***DGG***AY	1°7/13	γ	2	171	C_AR***S*****YRNHSR*****T***AY
2°/07^g^	γ	2	171	C_AR**DY*****GI***Y	3°/02	μ	2	171	C_AR***DGG***DY
2°/08	γ	2	171	C_AR***DGG***DY	3°/03^g^	μ	2	171	C_AR***DGGI***S
3°/05	μ	2	171	C_AR***DSG***DY	3°/04	μ	2	171	C_AR***A*****GRSY*****G***WYFDV
3°/19^g^	γ	2	171	C_AR***DGG***TY	3°/11^g^	γ	2	171	C_AR***DGG***AF
3°/20	γ	2	171	C_AR***DEGV***N					
**(B)**
1°7/03	μ	1	627	C_ARGYFDV	1°7/03	μ	1	286	C_AR***WGN***DY
1°7/04	μ	1	627	C_AR***A***NFDY	1°7/08	μ	6	114	C_TR***RG***TH
1°7/07	μ	1	396	C_***AIR***DY	1°7/11	γ	11	183	C_AR***NWG***DY
1°7/12	μ	3	128	C_ARRYFDV	1°14/01	μ	1	706	C_AR***R*****D**AY
1°7/25	γ	3	128	C_ARRYFDV	1°14/18	γ	1	528	C_AR***R***FAY
1°7/29	γ	5	139	C_AR***SPG***DY	2°/01	μ	6	114	C_TR***RG***DY
1°7/32	γ	14	125	C_***VPV***AWFAY					
1°14/03	μ	1	495	C_A***RWE***AY					
1°14/05	μ	1	073	C_AR***DGG***AY^h^					
3°/04	μ	1	286	C_AR***RDG***AY					
3°/08	μ	5	139	C_AR**DYG**DY^h^					
3°/09	μ	5	139	C_AR**DYG**DY^h^					
3°/10	μ	6	494	C_T***GG***PWFAY					
3°/18	γ	1	175	C_AR***DWGD***Y^h^					
3°/22	γ	5	139	C_AR**DYG**AY^h^					
3°/23	γ	6	114	C_T***TRG***DY					
3°/29	γ	14	125	C_AS**DYG*****L***Y					

*^a^The complete characteristics of these antibodies are compiled in Tables S1 and S2 in Supplementary Material*.

*^b^The designation of antibodies of ΔD-DμFS and ΔD-iD mutant mice indicates their generation on day 7 (1°7) or day 14 (1°14) after primary or on day 3 after secondary (2°) or tertiary (3°) immunization and is followed by a sequential number*.

*^c^Isotype of antibodies*.

*^d^Indicates the V_H_ gene family*.

*^e^V_H_ gene numbers according to the integrative database VBASE2*.

*^f^The CDR-H3 amino acid sequences are given in the one-letter code. Amino acids in bold are derived from the respective D_H_ gene segments while those in italics and underlined are generated by N region insertions and P nucleotides*.

*^g^V_H_ 171-encoded antibodies in combination with the Id_Ox1_ V_L_ gene Vκ072 are indicated by a gray background*.

*^h^These CDR-H3 amino acid sequences have already been observed in Id_Ox1_ antibodies from BALB/c WT mice*.

Short CDR-H3 with 6–8 amino acids (i.e., in the range of CDR-H3 of Id_Ox1_ antibodies) were also found in association with V_H_ genes other than V_H_171. These alternative V_H_ belonged to non-V_H_2 families (Table 2B). Two of the V_H_171-encoded antibodies exhibited longer CDR-H3. Thus, the great majority of anti-phOx mAbs produced in these D-altered mice used H chains that differed greatly from the characteristic Id_Ox1_ sequence, even though classic Id_Ox1_-like CDR-H3s could be generated by both D-altered mice.

It has been argued that diversity at CDR-H3 is sufficient for the creation of Ag-specificity and that a particular specificity can be correlated with identical or very similar amino acids sequences in CDR-H3 (16). Our anti-phOx mAb from the two D-limited mouse strains offered the opportunity to check this hypothesis in another experimental system. A survey of all anti-phOx antibodies from ΔD-DμFS and ΔD-iD mice is depicted in Tables S3A,B in Supplementary Material, respectively. From the 83 mAb of ΔD-DμFS mice, 58 (70%) made use of varying lengths of the genomic D_H_ sequence, 2 (2.4%) used inverted sequences, and in 23 (28%) the genomic D_H_ sequence was not detectable. From the 44 mAb of ΔD-iD mice, 23 (52%) used varying lengths of the D_H_ segment, 5 (11%) used variable lengths of the inverted sequence, and in 16 mAb (36%), no portion of the D_H_ segment could be identified. This large variability of CDR-H3-coding sequences led to an equal variability of 0–13 amino acid sequences in CDR-H3 loops. The average hydropathicity values of CDR-H3 loops varied from strongly hydrophobic to strongly hydrophilic (Table 3). Similar findings were obtained in WT mice (data not shown). Thus, since common sequences or sequence motifs in CDR-H3 could not be detected, we conclude that Ag-specificity in this model, immune response is not limited by either this parameter or by other, readily apparent physicochemical properties of the CDR-H3 interval.

**Table 3:** **Exemplary representation of the spectrum of CDR-H3 lengths and average hydropathicity values of CDR-H3 loops of selected anti-phOx antibodies from D-altered ΔD-DμFS and ΔD-iD mice**.

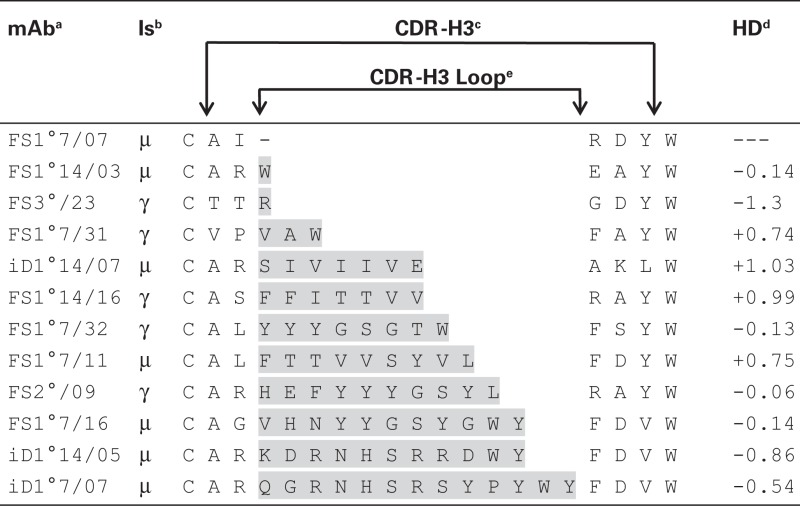

*^a^The complete characteristics of these antibodies are compiled in Tables S1 and S2 in Supplementary Material. The designation of antibodies of ΔD-DμFS (FS) and ΔD-iD (iD) mutant mice indicates their generation on day 7 (1°7) or day 14 (1°14) after primary or on day 3 after secondary (2°) or tertiary (3°) immunization and is followed by a sequential number*.

*^b^Isotype of antibodies*.

*^c^The CDR-H3 amino acid sequences are given in the one-letter code*.

*^d^Average hydropathicity of CDR-H3 loops*.

*^e^Amino acids of the CDR-H3 loops are accented by a gray background*.

## Discussion

In WT BALB/c, the preference for Id_Ox1_ anti-phOx Ab reflects both the failure of B cells producing phOx-binding IgM antibodies that use V_H_1-family genes to contribute to the IgG Ab repertoire and a focusing of CDR-H3 content (10). While the increase of Ab encoded by non-Id_Ox1_ V_H_/V_L_ gene combinations from later stages of the response may reflect enhanced K_a_ (17), the initial dominance of the Id_Ox1_ has been attributed to its superior *K*_D_ for the hapten phOx (14). This view has been challenged by the finding that the early primary IgM response contains a considerable number of anti-phOx Ab that demonstrate similar or even higher affinities than Id_Ox1_ Ab (10). Because these high affinity IgM progenitors do not have counterparts among IgG Ab (10), these findings have raised questions about a strict attribution of higher affinity for Ag as the primary force behind the clonal selection and CSR exhibited by B cells that express Ig bearing the Id_Ox1_ idiotype.

Attribution of the preference for the Id_Ox1_ idiotype as reflective of higher Ag affinity carries with it the presumption that the choice of specific Ab reflects selection from a random repertoire of antigen binding sites. However, although VDJ rearrangement and N addition yields a tremendously diverse CDR-H3 repertoire, inspection of the actual composition of the antigen binding site repertoire reveals a major bias in CDR-H3 amino acid content. Tyrosine and glycine are preferred, whereas charged and hydrophobic amino acids are under-represented. This non-random pattern of amino acid usage reflects natural selection for a bias in D gene segment sequence by reading frame that is coupled to a bias in reading frame choice (8). Together, these conserved biases yield a greatly restricted repertoire with some types of antigen binding sites represented more frequently than random chance would allow, and others grossly under-represented.

To test the functional significance of constraints introduced by natural selection of the germline antibody repertoire on B cell development and antibody production, we previously altered the D_H_ locus in BALB/c mice to force use of alternative D_H_ sequence (8). Violation of naturally selected germline constraints on CDR-H3 content led to impairments in total serum IgG levels, in tetanus-specific IgG antibody production after tetanus toxoid vaccination, and to the ability to protect against re-infection with influenza virus of a different serotype, termed heterosubtypic immunity. All of these facets of antibody production and protection are T cell dependent.

In the present work, we have gone beyond global measurements of total immunoglobulin production or protection against infection with a pathogen to assess the effects on T dependent antibody production at the individual B cell sequence level in the classic phOx system, whose study yielded many of the paradigms still in common thought to this day.

Here, we report that changes in naturally selected D_H_ sequence have not only yielded a decrease in serum immunoglobulin levels directed against yet another T cell dependent antigen; they have resulted in major changes at the molecular level to the nature of the antibody produced even though the simple addition of only five nucleotides of N addition can yield the classic I Id_Ox1_ irrespective of the sequence of the D. In particular, we observe enhanced and persistent production of hybridomas secreting low affinity IgM after secondary and tertiary challenge, as well as a failure to develop a fully mature, class switched IgG response equivalent to that produced by WT mice.

Classically, anti-phOx hybridomas obtained on day 3 of memory responses predominantly produce IgG mAb (13, 15). Therefore, we anticipated that the D-altered mice would also primarily produce IgG mAb. The consistently high rescue of IgM-producing hybridomas from memory responses was thus unexpected.

Development of a fully mature IgG response in WT mice is marked by clonal expansion of B cells using the V_H_Ox1/V_κ_Ox1 gene combination coupled with a focusing of CDR-H3 sequence with preference given to CDR-H3s encoded a short, DRG containing peptide sequence. In the D-altered mice, V_H_ and V_L_ variability persisted (Figure 2), V_H_1 family-encoded mAb continued to be produced, and no evidence of focusing of CDR-H3 sequence and structure was observed (Figures 2 and 3). This failure to clonally select for the various elements of the Id_Ox1_ idiotype occurred in spite of the fact that both mutant strains were able to generate an anti-phOx antibodies containing both the Id_Ox1_ V_H_171 H chain/Vκ072 L chain combination in conjunction with a CDR-H3 sequence similar or near identical to the classic idiotype (Table S3a in Supplementary Material).

Together, these findings suggest that the impairment in antibody production that we had observed in previous studies as a result of violation of evolutionarily conserved D gene sequence is accompanied by impairment of hallmarks of affinity maturation, such as clonal expansion and class switching. This occurs even when the classically preferred anti-phOx sequence can not only in theory be generated in the absence of D specific sequence, but is present in practice and thus available for clonal expansion and class switching.

The mechanism(s) that have led to the failure of antigen driven T cell dependent clonal expansion to produce the expected outcome are unclear. Two possible mechanisms involve B cells alone. First, the failure of the Id_Ox1_ idiotype to dominate could reflect the reduced likelihood of creating the Id_Ox1_ CDR-H3 sequence that might result from the loss of the D_H_ gene segments that normally contribute to its generation. However, comparable Id_Ox1_ CDR-H3 sequence was detected in the D-altered mice. Moreover, in WT mice the Id_Ox1_^+^ Ab begins as a minority of the early primary IgM response. It only dominates with the help of T cells, since it occurs only after class switching (Figure 2). Thus, attributing the change in outcome to an absence or diminution in the initial prevalence of Id_Ox1_ CDR-H3 is not compelling.

Second, the immunological imprinting that normally occurs during ontogeny might be altered as a result of the global change in the CDR-H3 repertoire (18, 19). The importance of controlling the B cell repertoire is underscored by the observation that neonatal injection or maternally derived anti-idiotypic antibodies may induce a drastic distortion of the adult B cell repertoire (20, 21) and maternal anti-idiotypes can even induce a long-lasting transgenerational suppression of IgE responsiveness (22). However, these types of early imprinted responses typically involve B cells expressing specificities directed against antigens encountered early in life. PhOx, however, is a foreign and manufactured antigen.

Alternatively, since the response that we have studied is T dependent, the mechanisms that have led to failure of clonal expansion and class switch recombination (CSR) could also reflect a D_H_ sequence dependent effect on interactions between T cells and B cells. There are several possible mechanisms by which this could occur. First, in the D-altered mice, we have previously observed changes in the distribution of B cell subset numbers and in the repertoire expressed by these B cells (8). It has been suggested that TD Ag-activated IgG^+^ and IgM^+^ memory B cells form a whole spectrum of memory B cell populations (23), although this view is not undissented (24). Most memory B cells appear to differentiate as a result of germinal center reactions. However, they can also be generated in a GC-independent manner in the follicles or even outside follicles (25). Many GC-independent follicular memory B cells are of the IgM^+^-only type (25–27), contain few or no somatic mutations and have not undergone affinity maturation (28–30). These same attributes are found in our memory IgM anti-phOx mAb (Figure 1). It is thus possible that the memory IgM mAb in the D-altred mice derive from a GC-independent pathway.

A second possibility is that the change in the repertoire of D sequence-associated antigen binding sites alters the antigen presentation properties of B cells for the phOx bearing antigen. D-alteration shifts the distribution of antigen binding sites, including enrichment for sites that are normally rare and depletion of sites that are normally common. The production of novel immunoglobulins with high affinity for the hapten could lead to changes in the peptides derived from the carrier protein that are presented to T cells by B cells in their capacity as antigen presenting cells (31). A global altered pattern of epitope recognition by responding T cells could inhibit T cell helper driven clonal expansion and affinity maturation.

A third possible T dependent mechanism could reflect the role of CDR-H3 as a potential T cell epitope, and thus contribute to distortions in T cell–B cell interactions (32–35). For example, the response to the TI-2 antigen α (1–3) dextran can be influenced by CDR-H3-specific T cells, which inhibit CSR of the dominant J558 IgM idiotype (36). And, BCR-specific T cells have been shown to be capable of interrupting an ongoing GC reaction, favoring the differentiation of short-lived extrafollicular plasmablasts (37). This interaction could help explain the enhanced yield of IgM hybridomas that we observed during memory responses in the D-altered mice, as well as explaining the reduced humoral IgM titers at later times of these responses (Figure 1). We would note that none of these potential mechanisms are mutually exclusive. Thus, one or more could be contributing to the failure of this TD response. Studies to clarify the mechanisms by which this has occurred are ongoing in our laboratory.

In this manuscript, we report a test of the hypothesis that conservation of the sequence signature of H chain diversity gene segments, which reflects the effect of natural selection, can influence the outcome of a T dependent response to antigen at the Ab molecular level. We found that subverting the effects of natural selection on the B cell CDR-H3 repertoire led to an alteration of the pattern of T cell-dependent CSR-associated clonal progression in these mutant mice (no dominant idiotypes, no elimination of V_H_1 family-encoded antibodies, and no selection of homogeneous CDR-H3). Our finding that the sequence of the D_H_ controls this specific TD response suggests that a full understanding of protective and non-protective responses to self or exogenous antigens, including vaccines, pathogens, and self antigens, will likely require clarification of the role of natural constraints on the antigen binding site repertoire. Vaccination strategies may need to be modified in order to take into account the constraints on humoral responses imposed by evolution.

## Conflict of Interest Statement

The authors declare that the research was conducted in the absence of any commercial or financial relationships that could be construed as a potential conflict of interest.

## Supplementary Material

The Supplementary Material for this article can be found online at http://www.frontiersin.org/Journal/10.3389/fimmu.2014.00385/abstract

## Abbreviations

CSA, chicken serum albumin; Id_Ox1_, Ox1 idiotype; phOx, 2-phenyloxazolone.
