# Supplementary material for: Clonal Progression during the T Cell-Dependent B Cell Antibody Response Depends on the Immunoglobulin DH Gene Segment Repertoire
Source: Front Immunol. 2014 Aug 11;5:385. doi: 10.3389/fimmu.2014.00385 (PMC4128299; doi:10.3389/fimmu.2014.00385)
Supplement: Supplementary file 1 [file DataSheet_1.DOCX]

**Supplementary materials**

**Clonal progression during the T cell-dependent**

**B cell antibody response depends on the**

**immunoglobulin D_H_ gene segment repertoire.**

**Ahmad Trad, Radu Iulian Tanasa, Hans Lange, Michael Zemlin, Harry W. Schroeder Jr. and Hilmar Lemke**

**Supplementary Figure 1**

Generation of a D-altered IgH allele


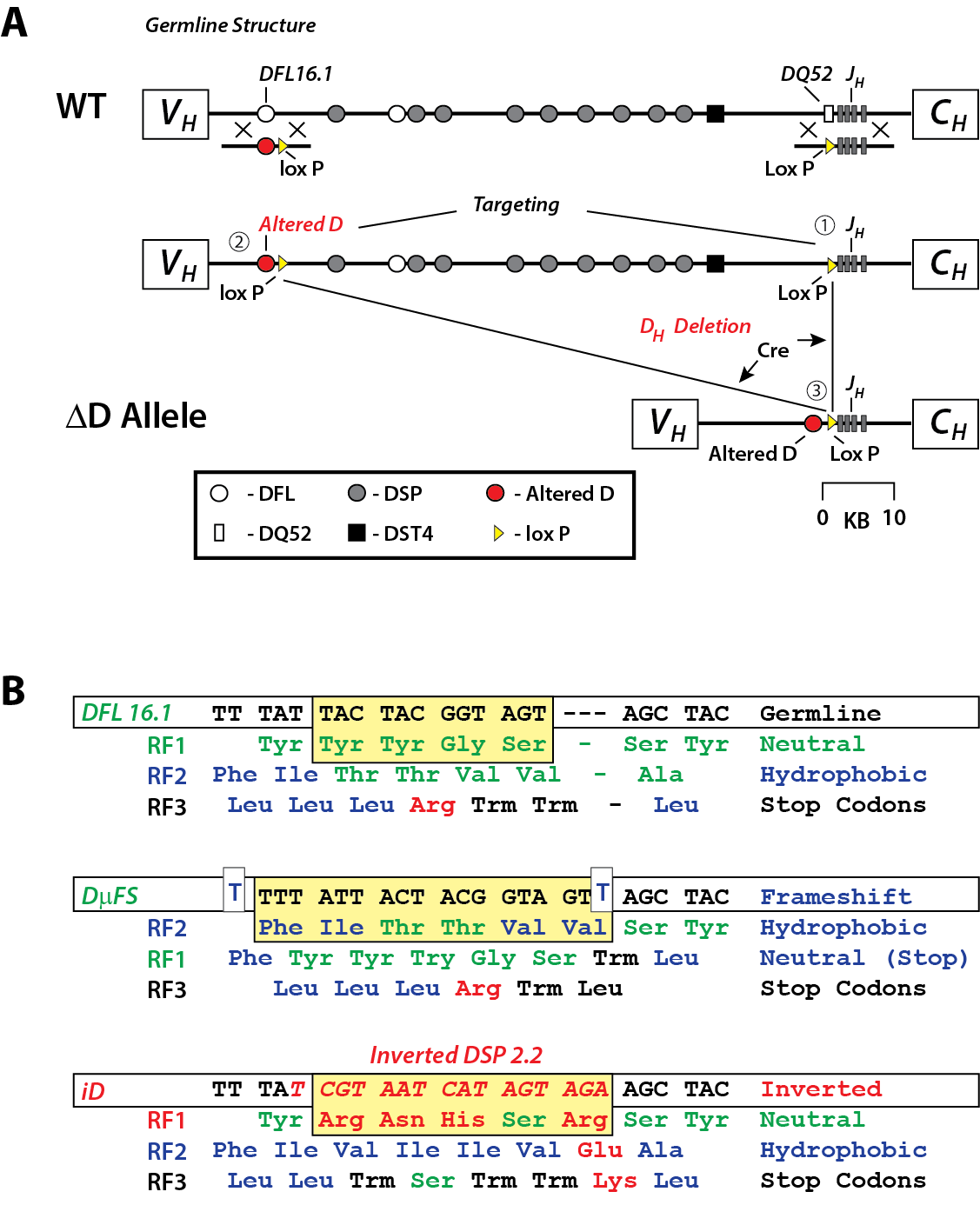


(A) Cre-loxP gene targeting and site-specific sequence modification by PCR was performed on the D_H_ locus BALB/c ES cell line (12). First, the J_H_ proximal DQ52 gene segment was replaced by a loxP site. Second, a lox P site was placed immediately 3’ to DFL16.1, the most V_H_ proximal D gene segment. Finally, the sequence between the two loxP sites, which contained 11 D_H_ gene segments belonging to the DFL, DSP and DST gene families, was deleted using cre. The targeted ES cells were injected into C57BL/6 blastocyst and the BALB/c offspring were bred to produce mice homozygous for the D_H_ alteration.

(B) The DμFS D_H_ gene segment (middle sequence) was created by inserting two T nucleotides and thus creating two separate frameshift mutations in the DFL16.1 gene segment (top sequence) (7). The first T shift the ATG start site upstream of the D_H_ into reading frame 1. The second T insertion shifts the 3’ terminal six nucleotides into reading frame 2, promoting microhomology-driven rearrangement into this reading frame in place of reading frame 1. A second effect of this insertion is to create a terminal codon in reading frame 1, further limiting its use. As a result, use of the reading frame encoding hydrophobic amino acids is now preferred.

The iD D_H_ gene segment (bottom sequence) was created by replacing the middle portion of the DFL16.1 gene segment with the inverted sequence of the DSP2.2 gene segment (6). DSP 2.2 inverted reading frame 2 has been placed in frame with the upstreatm ATG start site, limiting its use. The 5’ and 3’ sequence of the DFL16.1 gene segment have been preserved to promote microhomology-driven preferential rearrrangement into the reading frame encoding charged amino acids.

**Supplementary Figure 2**

D altered mice express polyclonal, altered CDR-H3 repertoires.


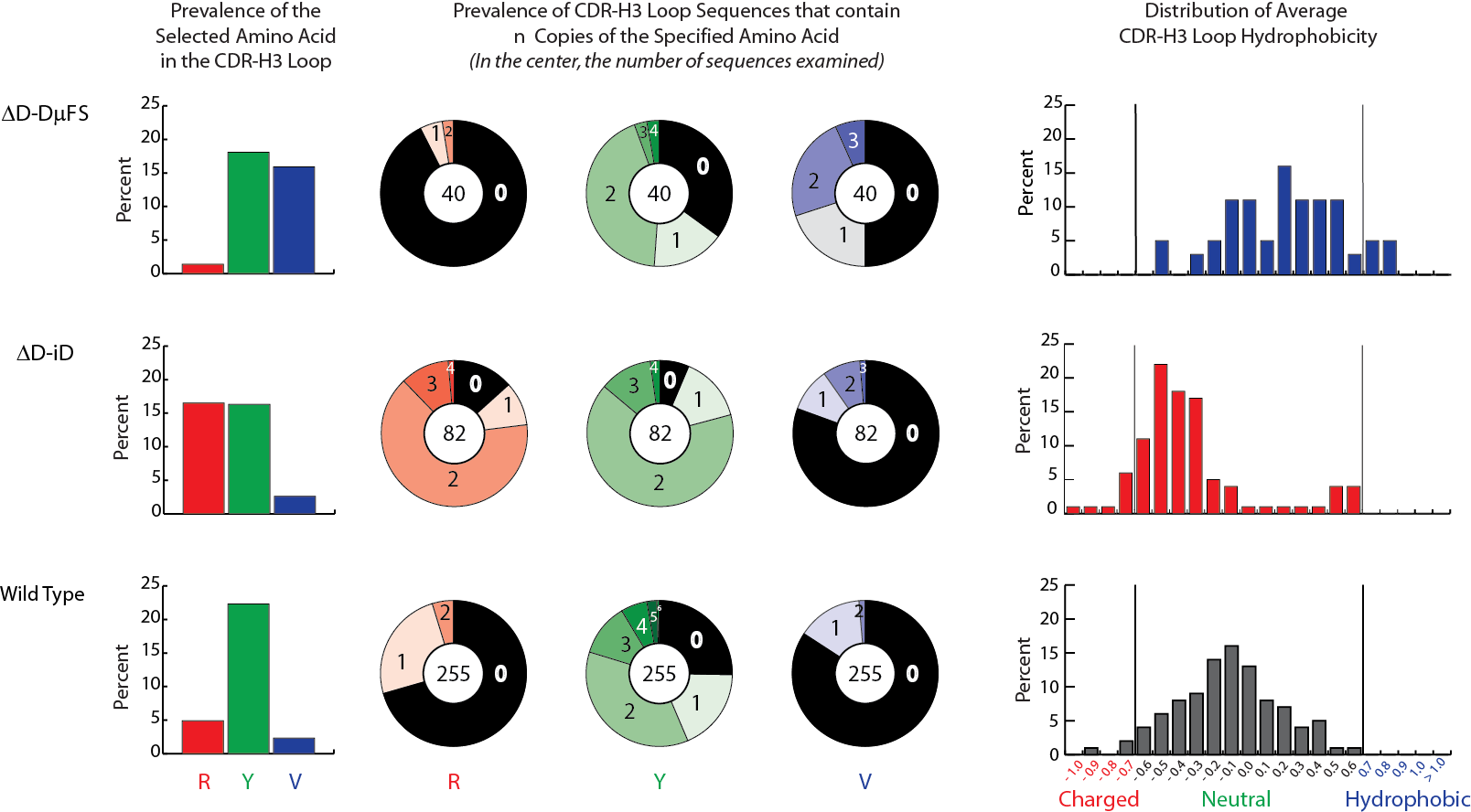


Tyrosine, valine and arginine are the most prominent amino acids populating D_H_ reading frame 1, reading frame 2, and inverted reading frame 1, respectively. Replacing the normal complement of D_H_ with a D engineered to favor the use of RF2 (DμFS) or a D engineered to favor the use of iRF1 results in a redistribution of the amino acid content and hydrophobicity of the CDR-H3 loop. V_H_7183DJC transcripts from sorted CD19^+^IgM^+^IgD^-^ immature B cells from the bone marrow of mice homozygous for the ΔD-DμFS (top) or ΔD-iD (middle) D_H_ alleles were cloned and the sequence of their CDR-H3 loops was compared to WT (bottom) littermate controls/

(Left) The prevalence of tyrosine, valine and arginine in the CDR-H3 loops. Relative to wild-type, CDR-H3 loops from ΔD-DμFS mice are enriched for valine whereas CDR-H3 loops from ΔD-iD mice are enriched for arginine. CDR-H3 loops from both D-altered mice are depleted of tyrosine.

(Middle) The percentage of CDR-H3s loop sequences that contain 1, 2, 3, 4, 5, or 6 tyrosine, valine or arginine amino acids. Almost half of CDR-H3 sequences from the ΔD-DμFS mice contain at least one valine. Almost 90% of the CDR-H3 sequences from the ΔD-iD mice contain at least one arginine.

(Right) Distribution of average hydrophobicity in the CDR-H3 loops. The normalized Kyte-Doolittle hydrophobicity scale has been used to calculate average hydrophobicity [reviewed in (8)]. To facilitate visualization of the change in distribution, the vertical lines mark the preferred range average hydrophobicity observed in wild-type CD19^+^IgM^+^IgD^+^ mature B cells from the bone marrow (8). CDR-H3 loops from the ΔD-DμFS mice tend to be more hydrophobic and CDR-H3 loops from the ΔD-iD mice tend to be more charged.

**Supplementary Figure 3A-H**


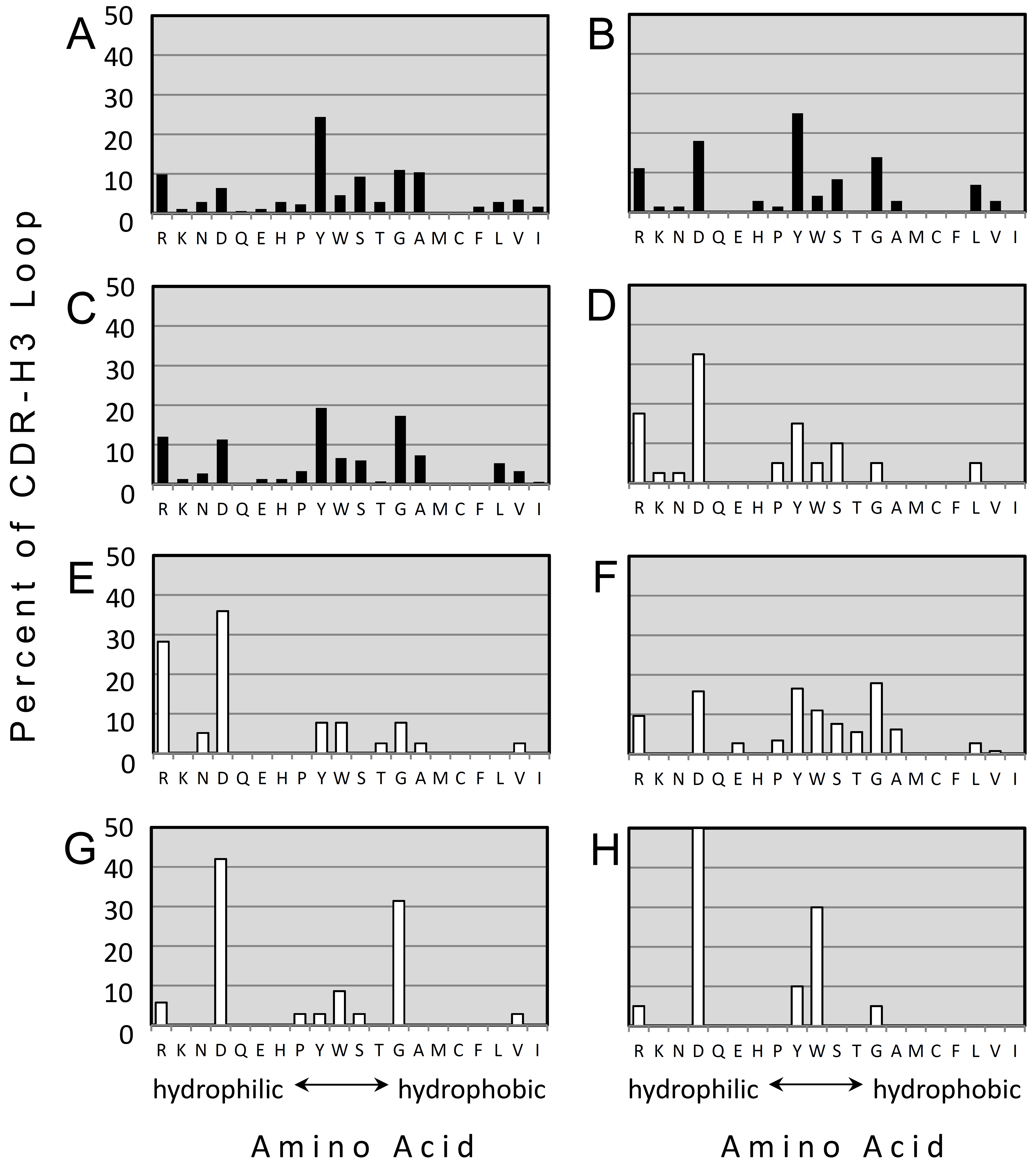


Amino acids of CDR-H3 loops of monoclonal anti-phOx antibodies of BALB/c wild-type mice ([1](#_ENREF_1), [2](#_ENREF_2))

Hybridomas secreting anti-phOx antibodies were generated from non-immunized mice (a) natural IgM (n = 30 mAb), (b) IgM from the primary response to TI-2 antigen phOx-Ficoll (n = 19 mAb), (c) IgM obtained on day 7 of the primary TD response to phOx-CSA (n = 36 mAb), (d) IgG of the phOx-CSA-induced TD response on day 7 (n = 13 mAb), (e) IgG from the late primary response on day 14 (n = 17 mAb), (f) phOx-CSA-induced secondary IgG (n = 30 mAb), (g) phOx-CSA-induced tertiary IgG (n = 16 mAb) and (h) TD-induced quaternary IgG (n = 10 mAb).

**Supplementary Figure 4A-H**


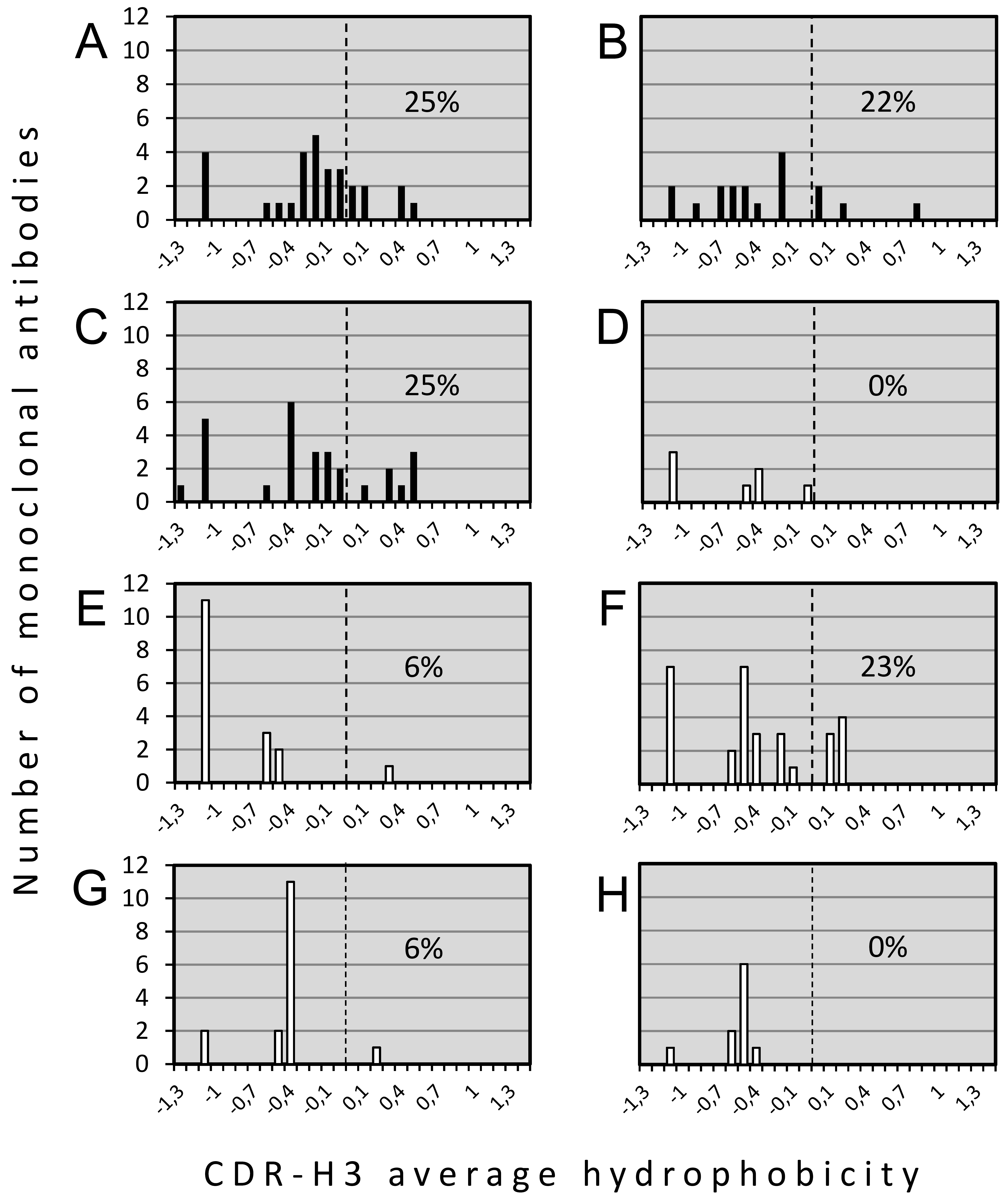


Distribution of average hydropathicity values of CDR-H3 loops of monoclonal anti-phOx antibodies of BALB/c wild-type mice ([1](#_ENREF_1), [2](#_ENREF_2))

The average hydropathicity values of CDR-H3 loops were calculated with the normalized Kyte-Doolittle hydrophobicity scale ([3](#_ENREF_3)). Graphs with black bars indicate IgM while those with white bars indicate IgG antibodies. The groups of antibodies and n-numbers correspond to those in Supplementary Figure 2. (a) natural IgM, (b) TI-2 antigen-induced primary IgM, (c) early day 7 primary IgM induced with the TD antigen phOx-CSA, (d) early primary TD-induced IgG, (e) late day 14 primary TD-induced IgG, (f) phOx-CSA-induced secondary IgG, (g) phOx-CSA-induced tertiary IgG and (h) TD-induced quaternary IgG. The proportion of antibodies with positive average hydropathicity values is indicated.

**Supplementary Table 1a**

V_H_/V_L_ gene combinations and CDR3 amino acid sequences of primary IgM anti-phOx antibodies from ΔD-DμFS mice obtained on day 7 after primary immunization with the thymus-dependent antigen phOx chicken serum albumin

**mAb**^a^ **Is**^b^ **rel. V_H_ chain genes V_L_ chain genes**

**FS1°7 Aff.**^c^ **Fam**^d^ **Cl**^e^ **IGHV**^f^ **CDR-H3**^g^ **RF**^h^ **J Fam**^d^ **Cl**^e^ **IGKV**^f^ **CDR-L3**^g^ **J**

01μ0.01 1 2 673C_ARGYFDVn.f.^i^ 1 9b 1 121C_LQYDEF***P***YTF2

02μ0.5 1 1 671C_**VITTVVS*L***V2 1 10 1 139C_QQYSKL***P***WTF1

03μ0.5 1 2 627C_ARGYFDVn.f. 1 4/5 1 072C_QQWSSN***P***FTF4

04μ0.5 1 2 627C_AR***A***NFDYn.f. 2 10 1 139C_QQYSKL***P***WTF1

05μ0.05 1 1 532C_AR***WGS*ITKVVS**VY2 2 4/5 1 078C_QQYSGY***PL***TF2

06μ1.0 1 2 506C_AS***V*YYYGR**DC1 2 9b 1 121C_LQYDEF***PR***TF1

07μ- 1 3 396C_***AIR***DYn.f. 2 19/28 1 201C_QQYNSY***PL***TF4

08μ0.1 1 3 386C_***AR*AT*AMG***AWFAY2 3 9a 1 112C_LQYANS***P***YTF2

09μ4.5 2 1 171C_AR***LTQT***FAYn.f. 3 not determined

10μ0.3 3 1 138C_AR**FTTVV*C***YWYFDV2 1 4/5 1 163C_QQWSGY***P***LTF5

11μ- 3 1 138C_A***L*FTTVVSY*VL***FDY2 2 19/28 1 175C_QQYSSY***P***WTF1

12μ0.05 3 1 128C_ARRYFDVn.f. 1 not determined

13μ5.0 3 1 120C_AR***V*YY*ED***GFAY1 3 21 1 066C_QHSWEI***PP***TF1

14μ- 6 1 114C_***AG*VV**RFAY2 3 9b 1 106C_LQHGES***P***YTF2

15μ2.0 9 1 155C_AR***D*YYY*EG***MDY1 4 1 1 122C_SQSTHV***PR***TF1

16μ0.5 12 1 117C_AG***VH*NYYGSY*G***WYFDV1 1 9b 1 121C_LQYDEF***P***YTF2

17μ- not determined4/5 1 154C_HQYHRS***PP***TF2

18μ- not determined9b 1 121C_LQYDEF***P***YTF2

Legend (next page)

Legend

^a^ The annotation of antibodies indicates their generation after primary immunization and fusion on day 7 followed by a sequential number (GenBank accession no. JX492322 – JX492353).

^b^ Isotype of anti-phOx antibodies

^c^ The relative affinities were measured in a hapten inhibition test in comparison to Id_Ox1_ prototypic anti-phOx IgM antibody H11.5.

^d^ Indicates the V_H_ gene family and the V_L_ subgroup, respectively.

^e^ In the integrative database VBASE2, the genes are classified: for class 1 genes there is genomic and rearranged evidence, for class 2 genes only genomic evidence and for class 3 genes only rearranged evidence.

^f^ VH and VL genes numbers according to the integrative database VBASE2.

^g^ The amino acid sequences of the third hypervariable regions of the heavy and light chains are given in the one-letter code. Amino acids in bold are derived from D gene-segments while those in italics and underlined are generated by N region insertions and P nucleotides.

^h^ Reading frame usage of CDR-H3.

^i^ n.f. – not found

**Supplementary Table 1b**

V_H_/V_L_ gene combinations and CDR3 amino acid sequences of primary IgG anti-phOx antibodies from ΔD-DμFS mice obtained on day 7 after primary immunization with the thymus-dependent antigen phOx chicken serum albumin

**mAb**^a^ **Is**^b^ **rel. V_H_ chain genes V_L_ chain genes**

**FS1°7 Aff.**^c^ **Fam**^d^ **Cl**^e^ **IGHV**^f^ **CDR-H3**^g^ **RF**^h^ **J Fam**^d^ **Cl**^e^ **IGKV**^f^ **CDR-L3**^g^ **J**

19γ2.0 1 2 690C_AS**VANYRSNK*RGE***NYi2 2 21 1 210C_QQSKEV***P***WTF1

20γ2.0 1 2 690C_A**VITTV*AP***DV2 1 24/25 1 123C_AQNLEL***P***YTF2

21γ0.5 1 2 643C_AR***R*VSY*G***NYFDY2 2 21 1 210C_QQSKEV***PP***TF1

22γ0.5 1 1 532C_A**NYYG*TS***FDY1 2 4/5 1 077C_QQYHSY***PP***TF2

23^i^γ0.5 2 1 171C_AR***DPG***AYn.f.^j^ 3 4/5 1 072C_QQWSSN***P***LTF5

24γ0.1 3 1 128C_ARRYFDVn.f. 1 not determined

25γ2.5 3 1 128C_AR***GL*LV**YWYFDV2 1 4/5 1 143C_QQRSSY***PP***LTF5

26γ13.0 5 1 163C_AS**HYYG*TP***FAY1 3 4/5 1 072C_QQWSSN***PP***TF2

27γ14.0 5 1 163C_AR***HN***NYAMDYn.f. 4 23 1 168C_QQSNTW***P***FTF4

28γ5.5 5 1 139C_AR***SPG***DYn.f. 2 2 1 099C_VQGTHF***P***YTF2

29γ1.0 6 1 114C_AR***PG*YGS*P***WFAY1 3 1 1 115C_FQGSHV***P***YTF2

30γ0.1 7 1 158C_AR**PTVVS*F***YAMDY2 4 21 1 210C_QQSKEV***P***YTF2

31γ- 14 1 125C_***VPV***AWFAYn.f. 3 9a 1 108C_LQYASY***PP***TF1

32γ2.0 14 1 125C_A**LYYYGS*G***TWFSY1 3 12/13 1 172C_QHHYG***IP***FTF4

33γ- not determined 4/5 1 072C_QQWSSN***P***FTF4

34 γ 0.1 not determined 4/5 1 078C_QQYSGY***PL***TF1

35γ115.0 not determined not determined

36α- 14 1 125C_TS***YRQA*YGS**SWFAY1 3 12/13 1 172C_QHHYGS***P***YTF2

Legend next page

Legend

^a^ The annotation of antibodies indicates their generation after primary immunization and fusion on day 7 followed by a sequential number (GenBank accession no. JX49 2354 – JX492385).

^b^ Isotype of anti-phOx antibodies

^c^ The relative affinities were measured in a hapten inhibition test in comparison to the Id_Ox1_ prototypic IgG anti-phOx antibody NQ2/16.2.

^d^ Indicates the V_H_ gene family and the V_L_ subgroup, respectively.

^e^ In the integrative database VBASE2, the genes are classified: for class 1 genes there is genomic and rearranged evidence, for class 2 genes only genomic evidence and for class 3 genes only rearranged evidence.

^f^ V_H_ and V_L_ genes numbers according to the integrative database VBASE2.

^g^ The amino acid sequences of the third hypervariable regions of the heavy and light chains are given in the one-letter code. Amino acids in bold are derived from D gene-segments while those in italics and underlined are generated by N region insertions and P nucleotides. Amino acids surrounded by a rectangle are encoded by an inverse sequence of the D segment.

^h^ Reading frame usage of CDR-H3. In case an inverse reading frame is used in CDR-H3, amino acids encoded by complementary stretches of the DH gene segment are surrounded by a rectangle.

^i^ The Id_Ox1_ gene combination VH171/Vκ072 is on gray background.

^j^ n.f. – not found

**Supplementary Table 1c**

V_H_/V_L_ gene combinations and CDR3 amino acid sequences of primary anti-phOx antibodies from ΔD-DμFS mice obtained on day 14 after primary immunization with the thymus-dependent antigen phOx chicken serum albumin

**mAb**^a^ **Is**^b^ **V_H_ chain genes V_L_ chain genes**

**FS1°14 Fam**^c^ **Cl**^d^ **IGHV**^e^ **CDR-H3**^f^ **RF**^g^ **J Fam**^c^ **Cl**^d^ **IGKV**^e^ **CDR-L3**^f^ **J**

01μ1 2 569C_AR***S*YYYGR*A***FDY1 2 4/5 1 077C_QQYHSY***P***YTF2

02μ1 1 532C_AR***G*YYGS**SWFAY1 3 2 1 097C_WQGTHF***PH***TF4

03μ1 2 495C_A***RWE***AYn.f.^h^ 3 4/5 1 166C_QQFTSS***P***WTF1

04μ1 2 286C_AR***S*DYGSY**DWYFDV1 1 4/5 1 166C_QQFTSS***P***YTF2

05μ1 2 073C_AR***DGG***AYn.f. 3 10 1 129C_QQGQSY***P***YTF2

06μ5 1 147C_AR***H*PVVS*M***YYFDY2 2 23 1 179C_QNGHSF***P***FTF4

07μ9 1 155C_AR***KG*ATV*TFRV***FAYi1 3 4/5 1 082C_QQRSSY***P***FTF4

08μ9 2 118C_AR**FMVV*WG***SMDY2 4 2 1 097C_WQGTHF***PH***TF1

09  not determined 4/5 1 166C_QQFTSS***P***YTF2

10γ1 1 480C_AR***SD*FIT*HA***WFAY2 3 1 1 115C_FQGSHV***P***LTF5

11γ2 1 171C_AR***DF*G*K***D1 3 4/5 1 142C_HQGNSI***P***FTF4

12γ5 1 192C_AR***LS*TT*A***DY2 2 not determined

13γ5 1 139C_A***K*DYG*VG*** 1 3 9a 1 103C_LQYASSFTF4

14γ7 1 168C_SR***G*HYYG**SWFAY1 3 1 1 115C_FQGSHV***P***YTF2

15γ7 1 158C_AR***G*DYYG*C***WFAY1 3 not determined

16γ14 1 125C_AS***F*FITTVVR**AY2 3 12/13 1 172C_QHHYST***P***YTF2

Legend (next page)

Legend

^a^ The annotation of antibodies indicates their generation after primary immunization and fusion on day 14 followed by a sequential number (GenBank accession no. JX402386 – JX492414).

^b^ Isotype of anti-phOx antibodies

^c^ Indicates the V_H_ gene family and the V_L_ subgroup, respectively.

^d^ In the integrative database VBASE2, the genes are classified: for class 1 genes there is genomic and rearranged evidence, for class 2 genes only genomic evidence and for class 3 genes only rearranged evidence.

^e^ V_H_ and V_L_ genes numbers according to the integrative database VBASE2.

^f^ The amino acid sequences of the third hypervariable regions of the heavy and light chains are given in the one-letter code. Amino acids in bold are derived from D gene-segments while those in italics and underlined are generated by N region insertions and P nucleotides. Amino acids surrounded by a rectangle are encoded by an inverse sequence of the D segment.

^g^ Reading frame usage of CDR-H3. In case an inverse reading frame is used in CDR-H3, amino acids encoded by complementary stretches of the DH gene segment are surrounded by a rectangle.

^h^ n.f. – not found

**Supplementary Table 1d**

V_H_/V_L_ gene combinations and CDR3 amino acid sequences of anti-phOx antibodies from ΔD-DμFS mice obtained on day 3 after secondary immunization with the thymus-dependent antigen phOx chicken serum albumin

**mAb**^a^ **Is**^b^ **rel. V_H_ chain genes V_L_ chain genes**

**FS2° Aff.**^c^ **Fam**^d^ **Cl**^e^ **IGHV**^f^ **CDR-H3**^g^ **RF**^h^ **J Fam**^d^ **Cl**^e^ **IGKV**^f^ **CDR-L3**^g^ **J**

01 - 1 2 623C_AR***GWS*ITTVVSY**AY2 3 1 1 115C_FQGSHV***P***YTF2

02μ- 1 2 591C_AR***ERSF*YYGR*EW***WFAY1 3 not determined

03 ** **- not determined2 1 097C_WQGTHF***PQ***TF1

04 ** **- not determined9b 1 121C_LQYDEF***P***YTF2

05^i^γ16 2 1 171C_AR***DSG***DYn.f.^j^ 4 4/5 1 072C_QQWSSN***P***LTF5

06γ127 2 1 171C_AR***DGG***AYn.f. 3 4/5 1 072C_QQWSTN***P***LTF5

07γ65 2 1 171C_AR**DY*GI***Y1 3 4/5 1 072C_QQWSSN***P***LTF5

08γ19 2 1 171C_AR***DGG***DYn.f. 4 10 1 137C_QQGNTL***P***YTF2

09γ ** **- 2 1 162C_AR***HE*FYYYGSYL*R***AY1 3 1 1 115C_FQGSHV***PR***TF1

10γ2 7 1 158C_AR***G*HYYG*T***WFAY1 3 1 1 115C_FQGSHV***P***YTF2

11γ10 14 1 125C_VR**YYYGR*P***DY1 2 10 1 137C_QQGNTL***PP***TF1

12γ- not determined19/28 1 202C_LQHWNY***P***YTF2

Legend

^a^ The annotation of antibodies indicates their generation after secondary immunization followed by a sequential number (GenBank accession no. JX492415 – JX492434).

^b^ Isotype of anti-phOx antibodies

^c^ The relative affinities were measured in a hapten inhibition test in comparison to the Id_Ox1_ prototypic IgG anti-phOx antibody NQ2/16.2.

^d^ Indicates the V_H_ or V_L_ gene family.

^e^ In the integrative database VBASE2, the genes are classified: for class 1 genes there is genomic and rearranged evidence, for class 2 genes only genomic evidence and for class 3 genes only rearranged evidence.

^f^ V_H_ and V_L_ genes numbers according to the integrative database VBASE2.

^g^ The amino acid sequences of the third hypervariable regions of the heavy and light chains are given in the one-letter code. Amino acids in bold are derived from D gene-segments while those in italics and underlined are generated by N region insertions and P nucleotides.

^h^ Reading frame usage of CDR-H3.

^i^ The Id_Ox1_ gene combination VH171/Vκ072 is on gray background.

^j^ n.f. – not found

**Supplementary Table 1e**

V_H_/V_L_ gene combinations and CDR3 amino acid sequences of IgM anti-phOx antibodies from ΔD-DμFS mice obtained on day 3 after tertiary immunization with the thymus-dependent antigen phOx chicken serum albumin

**mAb**^a^ **Is**^b^ **V_H_ chain genes V_L_ chain genes**

**FS3° Fam**^c^ **Cl**^d^ **IGHV**^e^ **CDR-H3**^f^ **RF**^g^ **J Fam**^c^ **Cl**^d^ **IGKV**^e^ **CDR-L3**^f^ **J**

01μ1 2 627C_AS***GG*TTV*A***FDY2 2 24/25 1 096C_MQHLEY***P***LTF5

02μ1 2 495C_A***R*DYG*KRG***GY1 2 not determined

03μ1 2 286C_AS**ISTVV*I*** 2 2 8 1 195C_QQHYST***P***LTF 5

04μ1 2 286C_AR***RDG***AYn.f.^h^ 3 9a 1 112C_LQYASS***P***YTF2

05μ2 1 171C_AR***DSG***DYn.f. 2 10 1 137C_QQGNTL***P***YTF2

06μ5 1 178C_**ITTVVS**YWYFDV2 1 21 1 210C_QQSKEV***PV***TF1

07μ5 1 176C_AR***GR*ATVVSY**AWFAY2 3 8 1 195C_QQHYST***P***YTF2

08μ5 1 139C_AR**DYG**DY1 2 4/5 1 072C_QQWSSN***P***LTF5

09μ5 1 139C_AR**DYG**DY1 2 1 1 231C_FQGSHV***P***FTF4

10μ6 2 494C_T***GG***PWFAYn.f. 3 2 1 099C_VQGTHF***PR***TF5

11μ7 1 158C_AR***GA*GS*G***AWFAY1 3 1 1 115C_FQGSHV***P***FTF2

12μnot determined10 1 137C_QQGNTL***P***YTF2

13μnot determined 1 1 115C_FQGSHV***PR***TF1

Legend

^a^ The annotation of antibodies indicates their generation after tertiary immunization followed by a sequential number (GenBank accession no. JX492435 – JX492457).

^b^ Isotype of anti-phOx antibodies

^c^ Indicates the V_H_ gene family and the V_L_ subgroup, respectively.

^d^ In the integrative database VBASE2, the genes are classified: for class 1 genes there is genomic and rearranged evidence, for class 2 genes only genomic evidence and for class 3 genes only rearranged evidence.

^e^ V_H_ and V_L_ genes numbers according to the integrative database VBASE2.

^f^ The amino acid sequences of the third hypervariable regions of the heavy and light chains are given in the one-letter code. Amino acids in bold are derived from D gene-segments while those in italics and underlined are generated by N region insertions and P nucleotides.

^g^ Reading frame usage of CDR-H3.

^h^ n.f. – not found

**Supplementary Table 1f**

V_H_/V_L_ gene combinations and CDR3 amino acid sequences of IgG anti-phOx antibodies from ΔD-DμFS mice obtained on day 3 after tertiary immunization with the thymus-dependent antigen phOx chicken serum albumin

**mAb**^a^ **Is**^b^ **V_H_ chain genes V_L_ chain genes**

**FS3° Fam**^c^ **Cl**^d^ **IGHV**^e^ **CDR-H3**^f^ **RF**^g^ **J Fam**^c^ **Cl**^d^ **IGKV**^e^ **CDR-L3**^f^ **J**

14γ1 2 627C_AR***F*YYGR*GGLV***YFDY1 2 9a 1 103C_LQDGSS***PN***TF2

15γ1 2 627C_AR***F*YYGR*GGLV***YFDY1 2 12/13 1 174C_QHFWST***P***LTF5

16γ1 2 073C_AR***GG*FIRTTV*AG***DY2 2 4/5 1 078C_QQYSGY***P***LTF5

17γ1 2 073C_TT***G*DYYGS**Y1 2 not determined

18γ2 1 175C_AR***DWGD***Yn.f.^h^ 3 4/5 1 072C_QQWNSY***PPI***TF5

19^i^γ2 1 171C_AR***DGG***TYn.f. 3 4/5 1 072C_QQWSSN***P***LTF5

20γ2 1 171C_AR***DEGV***Nn.f. 3 10 1 137C_QQGKTL***P***YTF2

21γ3 1 138C_AR**FTTVVS**YWYFDV2 1 24/25 1 123C_AQNLEL***PP***TF1

22γ5 1 139C_AR**DYG**AY1 3 4/5 1 072C_QQWSGN***P***LTF5

23γ6 1 114C_T***TRG***DYn.f. 2 9a 1 108C_LQYASY***P***FTF2

24γ7 2 663C_AR***G*DYYG*A***WFAY1 3 1 1 115C_FQGSHV***P***FTF4

25γ7 1 158C_AR***G*DG*RG***AWFAY1 3 1 1 115C_FQGSHV***P***YTF2

26γ7 1 158C_AR***G*HYYG**AWFAY1 3 1 1 115C_FQGSHV***P***FTF4

27γ7 1 158C_AR***G*HYYG*T***WFAY1 3 1 1 115C_FQGSHV***P***FTF2

28γ7 1 158C_AR***G*DYYG**AWFAY1 3 1 1 115C_FQGSHV***P***FTF4

29γ14 1 125C_AS**DYG*L***Y1 2 10 1 129C_QQGQSY***PL***TF1

30γ14 1 125C_AR***YE*ITTVV*TSI*** 2 2 12/13 1 172C_QHHYGT***P***FTF4

31γnot determined4/5 1 078C_QQYSGY***PL***TF1

32γnot determined10 1 137C_QQGKTL***P***YTF2

Legend next page

Legend

^a^ The annotation of antibodies indicates their generation after tertiary immunization followed by a sequential number (GenBank accession no. JX492458 – JX492492).

^b^ Isotype of anti-phOx antibodies

^c^ Indicates the V_H_ gene family and the V_L_ subgroup, respectively.

^d^ In the integrative database VBASE2, the genes are classified: for class 1 genes there is genomic and rearranged evidence, for class 2 genes only genomic evidence and for class 3 genes only rearranged evidence.

^e^ V_H_ and V_L_ genes numbers according to the integrative database VBASE2.

^f^ The amino acid sequences of the third hypervariable regions of the heavy and light chains are given in the one-letter code. Amino acids in bold are derived from D gene-segments while those in italics and underlined are generated by N region insertions and P nucleotides.

^g^ Reading frame usage of CDR-H3.

^h^ n.f. – not found

^i^ The Id_Ox1_ gene combination VH171/Vκ072 is on gray background.

**Supplementary Table 2a**

V_H_/V_L_ gene combinations and CDR3 amino acid sequences of primary IgM anti-phOx antibodies from ΔD-iD mice obtained on day 7 after primary immunization with the thymus-dependent antigen phOx chicken serum albumin

**mAb**^a^ **Is**^b^ **rel. V_H_ chain genes V_L_ chain genes**

**iD1°7 Aff.**^c^ **Fam**^d^ **Cl**^e^ **IGHV**^f^ **CDR-H3**^g^ **RF**^h^ **J Fam**^d^ **Cl**^e^ **IGKV**^f^ **CDR-L3**^g^ **J**

01μ- 1 1 532C_AR***RG*RNHSRSY*VG***GYFDV1 1 2 1 097C_WQGTHF***PR***TF1

02μ0.05 1 1 532C_AR**IY*LI***PY1 3 23 1 171C_QQSNSW***P***YTF2

03μ0.05 1 2 286C_AR***WGN***DYn.f. 2 1 1 122C_SQSTHV***P***YTF2

04μ0.2 2 1 171C_AR***DRG***DYn.f. 2 4/5 1 078C_QQYSGY***PL***TF2

05μ0.1 2 1 171C_SR***DRG***DYn.f. 2 10 1 137C_QQGNTL***P***WTF1

06μ0.1 2 1 171C_AR***DRG***AYn.f. 3 21 1 210C_QQSKEV***P***YTF2

07μ0.05 5 1 131C_AR***QG*RNHSRSY*P***YWYFDV1 1 1 1 122C_SQSTHV***P***YTF2

08μ0.05 6 1 114C_TR***RG***THn.f. 3 9a 1 108C_LQYASY***P***YTF2

09μ- not determined8 1 195C_QQHYST***PF***TF5

10μ- not determined21 1 064C_QQSNED***P***FTF4

11γ- 2 1 183C_AR***NWG***DYn.f. 4 4/5 1 072C_HQWSSN***P***LTF5

12γ4.0 2 1 171C_AR***DRG***DYn.f. 4 1 1 115C_FQGSHV***P***YTF2

13γ5.5 2 1 171C_AR***S*YRNHSR*T***AY1 3 4/5 1 154C_HQYHRS***PP***TF5

14γ9.0 5 1 176C_A**SSFYYDYDK*S***FAYi2 3 8 1 195C_QQHYST***PP***F1

15γ1.0 5 1 139C_AR***P*VI*R***YYYAMDY2 4 4/5 1 050C_QQWSSN***P***YTF2

16γ1.0 6 1 114C_TR***P*LIV*RVH***FAY2 3 8 1 188C_QNDYSY***P***FTF4

17γ- not determined19/28 1 186C_QQDYSS***P***YTF2

Legend (next page)

Legend

^a^ The annotation of antibodies indicates their generation after primary immunization and fusion on day 7 followed by a sequential number (GenBank accession no. JX492493 – JX492523).

^b^ Isotype of anti-phOx antibodies

^c^ The relative affinities were measured in a hapten inhibition test in comparison to the Id_Ox1_ prototypic anti-phOx antibody H11.5 for IgM and NQ2/16.2 for IgG antibodies.

^d^ Indicates the V_H_ gene family and the V_L_ subgroup, respectively.

^e^ In the integrative database VBASE2, the genes are classified: for class 1 genes there is genomic and rearranged evidence, for class 2 genes only genomic evidence and for class 3 genes only rearranged evidence.

^f^ V_H_ and V_L_ genes numbers according to the integrative database VBASE2.

^g^ The amino acid sequences of the third hypervariable regions of the heavy and light chains are given in the one-letter code. Amino acids in bold are derived from D gene-segments while those in italics and underlined are generated by N region insertions and P nucleotides. Amino acids surrounded by a rectangle are encoded by an inverse sequence of the D segment.

^h^ Reading frame usage of CDR-H3. In case an inverse reading frame is used in CDR-H3, amino acids encoded by complementary stretches of the DH gene segment are surrounded by a rectangle.

^i^ n.f. – not found

**Supplementary Table 2b**

V_H_/V_L_ gene combinations and CDR3 amino acid sequences of primary anti-phOx antibodies from ΔD-iD mice obtained on day 14 after primary immunization with the thymus-dependent antigen phOx chicken serum albumin

**mAb**^a^ **Is**^b^ **V_H_ chain genes V_L_ chain genes**

**iD1°14 Fam**^c^ **Cl**^d^ **IGHV**^e^ **CDR-H3**^f^ **RF**^g^ **J Fam**^c^ **Cl**^d^ **IGKV**^e^ **CDR-L3**^f^ **J**

01μ1 1 706C_AR***RDA***Yn.f.^h^ 2 4/5 1 142C_QQGSSI***PR***TF2

02μ1 2 286C_AR**HHSRS**YWYFDV1 1 19/28 1 201C_QQYNSY***PR***TF1

03μ1 3 014C_***ARR*DRNHSRS*FL***YWYFDV1 1 10 1 137C_QQGNTL***PR***TF1

04μ3 1 128C_AR***G*VIILLA*R***YFDY2 2 4/5 1 050C_QQWSSN***PPI***TF4

05μ3 1 128C_AR***K*DRNHSRR*D***WYFDV1 1 10 1 137C_QQGNTL***P***FTF4

06μ5 1 192C_AR***P*FYRNHR*G***YFDY1 2 23 1 168C_QQSNSW***PT***WTF1

07μ5 1 185C_AR***S*IVIIVEA*KL*** 2 2 4/5 1 082C_QQRSSY***PP***LTF5

08μ5 1 178C_TR**VASTMITVK*RV***LDYi1 2 8 1 069C_QNDHSY***P***LTF5

09μ5 1 147C_AR***HP*L*RR***YFDY3 2 38c 1 140C_LQYDNL***R***TF5

10μ5 1 135C_A**NRNHSRS**YWYFDV1 1 4/5 1 072C_QQWSSN***PP***TF5

11μ5 1 135C_AR***PG*RNHSRSY*P***YFDY1 2 23 1 179C_QNGHSF***P***LTF5

12μ5 1 135C_AR***HR*RNHSRR*ET***DWYFDV1 1 23 1 168C_QQSNSW***P***FTF4

13μ6 1 114C_TR***PVIPE***AWFAYn.f. 3 1 1 115C_FQGSHV***P***WTF1

14μ10 1 028C*_*VR***HG*TYS*WR***DY3 2 12/13 1 170C_QHFWGT***P***LTF5

15μnot determined 12/13 1 170C_QHFWGT***P***WTF1

16μnot determined 2 1 097C_WQGTHF***P***TF1

17μnot determined 4/5 1 078C_QQYSGY***P***LTF5

18γ1 1 528C_AR***R***FAYn.f. 3 not determined

19γ1 2 455C_TR***SWG*DHS*KTYG***SWFAY1 3 1 1 115C_FQGSHV***PR***TF1

Legend (next page)

Legend

^a^ The annotation of antibodies indicates their generation after primary immunization and fusion on day 14 followed by a sequential number (GenBank accession no. JX492524 – JX492557).

^b^ Isotype of anti-phOx antibodies

^c^ Indicates the V_H_ gene family and the V_L_ subgroup, respectively.

^d^ In the integrative database VBASE2, the genes are classified: for class 1 genes there is genomic and rearranged evidence, for class 2 genes only genomic evidence and for class 3 genes only rearranged evidence.

^e^ V_H_ and V_L_ genes numbers according to the integrative database VBASE2.

^f^ The amino acid sequences of the third hypervariable regions of the heavy and light chains are given in the one-letter code. Amino acids in bold are derived from D gene-segments while those in italics and underlined are generated by N region insertions and P nucleotides. Amino acids surrounded by a rectangle are encoded by an inverse sequence of the D segment.

^g^ Reading frame usage of CDR-H3. In case an inverse reading frame is used in CDR-H3, amino acids encoded by complementary stretches of the DH gene segment are surrounded by a rectangle.

^h^ n.f. – not found

**Supplementary Table 2c**

V_H_/V_L_ gene combinations and CDR3 amino acid sequences of primary anti-phOx antibodies from ΔD-iD mice obtained on day 3 after secondary immunization with the thymus-dependent antigen phOx chicken serum albumin

**mAb**^a^ **Is**^b^ **rel. V_H_ chain genes V_L_ chain genes**

**iD2° Aff.**^c^ **Fam**^d^ **Cl**^e^ **IGHV**^f^ **CDR-H3**^g^ **RF**^h^ **J Fam**^d^ **Cl**^e^ **IGKV**^f^ **CDR-L3**^g^ **J**

01μ0.5 6 1114C_TR***RG***DYn.f.^i^ 2 9a 1 108C_LQYASY***PF***TF5

02γ300.0 1 3386C_AR***L*RNH*G***DS1 5 9a 1 108C_LQYASY***PP***TF2

Legend

^a^ The annotation of antibodies indicates their generation secondary immunization followed by a sequential number (GenBank accession no. JX492558 – JX492561).

^b^ Isotype of anti-phOx antibodies

^c^ The relative affinities were measured in a hapten inhibition test in comparison to the Id_Ox1_ prototypic anti-phOx antibodies H11.5 for IgM and NQ2/16.2 for the IgG antibody.

^d^ Indicates the V_H_ gene family and the V_L_ subgroup, respectively.

^e^ In the integrative database VBASE2, the genes are classified: for class 1 genes there is genomic and rearranged evidence, for class 2 genes only genomic evidence and for class 3 genes only rearranged evidence.

^f^ V_H_ and V_L_ genes numbers according to the integrative database VBASE2.

^g^ The amino acid sequences of the third hypervariable regions of the heavy and light chains are given in the one-letter code. Amino acids in bold are derived from D gene-segments while those in italics and underlined are generated by N region insertions and P nucleotides.

^h^ Reading frame usage of CDR-H3.

^i^ n.f. – not found

**Supplementary Table 2d**

V_H_/V_L_ gene combinations and CDR3 amino acid sequences of primary anti-phOx antibodies from ΔD-iD mice obtained on day 3 after tertiary immunization with the thymus-dependent antigen phOx chicken serum albumin

**mAb**^a^ **Is**^b^ **rel. V_H_ chain genes V_L_ chain genes**

**iD3° Aff.**^c^ **Fam**^d^ **Cl**^e^ **IGHV**^f^ **CDR-H3**^g^ **RF**^h^ **J Fam**^d^ **Cl**^e^ **IGKV**^f^ **CDR-L3**^g^ **J**

01μ- 2 1 201C_AK***MG*RNHSRS**YFDY1 2 4/5 1 050C_QQWSSNLTF5

02μ0.3 2 1 171C_AR***DGG***DYn.f. 2 10 1 137C_QQGNTL***P***YTF 2

03^i^1.0 2 1 171C_AR***DGGI***Sn.f. 3 4/5 1 072C_QQWSSN***P***LTF5

04μ- 2 1 171C_AR***A*GRSY*G***WYFDV1 1 4/5 1 079C_QQWSSY***PP***TF2

05μ0.03 5 1 192C_AR***SR*YD**YYAMDYi2 4 4/5 1 157C_QQWSSN***PPI***TF5

06μ- 5 1 147C_AR***I*NRNHSRSY*PG***YFDY1 2 10 1 137C_QQGNTLWTF1

07μ0.01 5 1 135C_AS**IYRNHSRS**HWYFDV1 1 21 1 210C_QQSKEV***P***WTF1

08μ- not determined 4/5 1 072C_QQWSSN***P***YTF2

09γ4.0 1 3 386C_VR***KFRGG***DYn.f. 2 9a 1 108C_LQYASY***P***YTF2

10γ3.0 1 3 386C_VR***KFRGG***DYn.f. 2 9a 1 108C_LQYGSY***P***YTF4

11^i^γ144.0 2 1 171C_AR***DGG***AFn.f. 3 4/5 1 072C_QQWSSN***P***LTF5

12γ14.0 3 1 120C_VR***GGTV*VA**FDYi1 2 23 1 168C_QENYSW***P***FTF4

13γ4.5 7 1 158C_AR***GL*YE*G***AWFSYi2 3 1 1 115C_FQGSHV***P***YTF2

14γ2.5 not determined 23 1 168C_QQSNSW***P***FTF2

15γ4.5 not determined RF 1 128C_QQHNEY***P***WTF1

Legend

^a^ The annotation of antibodies indicates their generation after tertiary immunization followed by a sequential number (GenBank accession no. JX492562 – JX492588).

^b^ Isotype of anti-phOx antibodies

^c^ The relative affinities were measured in a hapten inhibition test in comparison to the Id_Ox1_ prototypic anti-phOx antibodies H11.5 for IgM and NQ2/16.2 for IgG antibodies.

^d^ Indicates the V_H_ gene family or the V_L_ subgroup, respectively.

^e^ In the integrative database VBASE2, the genes are classified: for class 1 genes there is genomic and rearranged evidence, for class 2 genes only genomic evidence and for class 3 genes only rearranged evidence.

^f^ V_H_ and V_L_ genes numbers according to the integrative database VBASE2.

^g^ The amino acid sequences of the third hypervariable regions of the heavy and light chains are given in the one-letter code. Amino acids in bold are derived from D gene-segments while those in italics and underlined are generated by N region insertions and P nucleotides. Amino acids surrounded by a rectangle are encoded by an inverse sequence of the D segment.

^h^ Reading frame usage of CDR-H3. In case an inverse reading frame is used in CDR-H3, amino acids encoded by complementary stretches of the DH gene segment are surrounded by a rectangle.

^i^ The Id_Ox1_ gene combination VH171/Vκ072 is on gray background.

**Supplementary Table 3a**

CDR-H3 nucleotide sequences of anti-phOx antibodies from D-altered ΔD-DμFS mice

**V_H_ gene**^c^

**mAb**^a^ **Is**^b^F no. 3’-end  **N/P**^d^ **D_H_ segment-encoded**^e^ **N/P**^d^ **J_H_**^f^ **RF**^g^

**D_H_ of ΔD-DμFS mice: TTTTATTACTACGGTAGTTAGCTAC**

2°/09γ2 162 TGTGCCAGA ***CATGAA*** **TTTTATTACTACGGTAGTTACCTAC** ***GC*** GCTTACTGG 3 1

2°/01  1 623 TGTGCAAGA ***GGGTGGAG*** **TATTACTACGGTAGTTAGCTAC** - GCTTACTGG 3 2

1°14/16 γ 14 125 TGTGCTAG ***CTT*** **TTTTATTACTACGGTAGTTAG** ***G*** GCTTACTGG 3 2

1°7/11  3 138 TGTGCA ***CTCT*** **TTACTACGGTAGTTAGCTAC** ***GTACTT*** TTTGACTACTGG 2 2

1°7/02  1 671 TG ***CG*** **TTATTACTACGGTAGTTAGC** ***CT*** TGTCTGG 1 2

3°/16 γ 1 073 TGTGCAAGA ***GGAGGC*** **TTTATTAGGACGACGGTAG** ***CCGGG*** GACTACTGG 2 2

1°7/05  1 532 TGTGCAAGA ***TGGGGCTC*** **TATTACTAAGGTAGTTAG** - TGTCTACTGG 2 2

1°7/32 γ 14 125 TGTGCT ***C*** **TTTATTACTACGGTAGT *GGGA*** CCTGGTTTTCTTACTGG 3 1

3°/07  5 176 TGTGCAAGA ***GGCAGGG* CTACGGTAGTTAGCTAC *G*** CCTGGTTTGCTTACTGG 3 2

1°7/16  12 117 TGTGCAGGA ***GTCCATA*** **ATTACTACGGTAGTTA *CGGG*** TGGTACTTCGATGTCTGG 1 1

3°/06  5 178 TGTA - **TTACTACGGTAGTTAG** -CTACTGGTACTTCGATGTCTGG 1 2

3°/30 γ 14 125 TGTGCTAGA ***TATGAG*** **ATTACTACGGTAGTTA *CCTCTAT*** CTGG 2 2

3°/21 γ 3 138 TGTGCAAGA ***T*** **TTACTACGGTAGTTAG** -CTACTGGTACTTCGATGTCTGG 1 2

1°7/20 γ 1 690 TGTGCA ***G* TTATTACTACGGTAG *CCCC*** CGATGTCTGG 2 2

1°7/30 γ 7 158 TGTGCAAGA ***C* CTACGGTAGTTAGCT *T*** CTATGCTATGGACTACTGG 4 2

3°/03  1 286 TGTGCAAG - **TATTAGTACGGTAGT *CAT***  CTGG 2 2

1°7/06  1 506 TGTGCAAG ***TGTG* TATTACTACGGTAG *A*** GACTGCTGG 2 1

1°7/10  3 138 TGTGCAAGA ***T* TTACTACGGTAGTT** ***TG*** CTACTGGTACTTCGATGTCTGG 1 2

1°14/01  1 569 TGTGCAAGA ***TCC* TATTACTACGGTAG *GGC*** CTTTGACTACTGG 2 1

2°/11 γ 14 125 TGTGTTAGA - **TATTACTACGGTAG *GCC*** TGACTACTGG 2 1

1°14/04  1 286 TGTGCAAGA ***TCAG* ACTACGGTAGTTA *CG*** ACTGGTACTTCGATGTCTGG 1 1

3°/17 γ 1 073 TGTACAACA ***GGGG*** **ATTACTACGGTAG** -TTACTGG 2 1

1°7/22 γ 1 532 TGTGCAA - **ATTACTACGGTA *CCTC*** CTTTGACTACTGG 2 1

1°7/26 γ 5 163 TGTGCAAG ***CC* ATTACTACGGTA *CCCCT*** TTTGCTTACTGG 3 1

1°14/02  1 532 TGTGCAAGA ***GG* TTACTACGGTAG *CT*** CCTGGTTTGCTTACTGG 3 1

1°14/08  9 118 TGTGCCAGA - **TTTAT**___**GGTAGTT**^h^  ***TGGGGTT*** CTATGGACTACTGG 4 2

2°/02  1 591 TGTGCAAGA ***GAGAGGTCTTT* TTACTACGGTAG *GGAGTGG*** TGGTTTGCTTACTGG 3 1

2°/10 γ 7 158 TGTGCACGA ***GGGC* ATTACTACGGTA *CT*** TGGTTTGCTTACTGG 3 1

3°/27 γ 7 158 TGTGCAAGA ***GGTC* ATTACTACGGTA *CT*** TGGTTTGCTTACTGG 3 1

3°/15^i^ γ 1 627 TGTGCAAG ***GTT* TTACTACGGTAG *GGGGGGGCTCGT*** CTACTTTGACTACTGG 2 1

3°/14^i^ γ 1 627 TGTGCAAG ***GTT* TTACTACGGTAG *GGGGGGGCTCGT*** CTACTTTGACTACTGG 2 1

1°7/15  9 155 TGTGCAAGA ***GA* TTATTACTACG *AGGG*** TATGGACTACTGG 4 1

1°7/36 α 14 125 TGTACTAG ***CTACCGGCAGGC* CTACGGTAGTT** -CCTGGTTTGCTTACTGG 3 1

1°14/06  5 147 TGTGCAAGA ***CATC* CGGTAGTTAGC *ATGT*** ACTACTTTGACTACTGG 2 2

1°14/15 γ 7 158 TGTGCAAGA ***GGGG* ATTACTACGGT *TGT*** TGGTTTGCTTACTGG 3 1

3°/01  1 627 TGTGCAAG ***CGGGGG* TACTACGGTAG *C*** CTTTGACTACTGG 2 2

3°/24^k^ γ7 663TGTGCAAGA ***GGGG* ATTACTACGGT *G*** CCTGGTTTGCTTACTGG 3 1

3°/28^k^ γ 7 158 TGTGCAAGA ***GGGG*** **ATTACTACGGT *G*** CCTGGTTTGCTTACTGG 3 1

3°/26 γ 7 158 TGTGCAAGA ***GGGC* ATTACTACGGT *G*** CCTGGTTTGCTTACTGG 3 1

1°7/21 γ 1 643 TGTGCAAGA ***AG* AGTTAGCTAC G*GAA*** ACTACTTTGACTACTGG 2 2

1°14/10 γ 1 480 TGTGCAAGA ***TCTGA* TTTTATTACT *CACG*** CCTGGTTTGCTTACTGG 3 2

1°14/14 γ 7 168 TGTTCAAGA ***GGCC* ATTACTACGG *CT*** CCTGGTTTGCTTACTGG 3 1

1°7/29 γ 6 114 TGTGCCAGG ***CCGGGT* TACGGTAGT *C*** CCTGGTTTGCTTACTGG 3 1

1°7/13  3 120 TGTGCAAGA ***GT* TTACTACG *AAGACG*** GGTTTGCTTACTGG 3 1

1°14/12 γ 5 192 TGTGCAAGA ***CTGAG* TACTACGG *CG*** GACTACTGG 2 2

1°14/13 γ 5 139 TGTGCAA ***AAG* ACTACGGT *GTCGGG*** TGG 3 1

3°/08  5 139 TGTGCAAGA ***G* ACTACGGT** -GACTACTGG 2 1

3°/09  5 139 TGTGCAAGA ***G* ACTACGGT** -GACTACTGG 2 1

1°7/14  6 114 TGT ***GCGGGC* GTAGTTA** -GGTTTGCTTACTGG 3 2

3°/02  1 495 TGTGCAA ***GAG* ACTACGG *CAAAAGAGG*** TGGCTACTGG 2 1

3°/22 γ 5 139 TGTGCAAGA ***G* ACTACGG *G*** GCTTACTGG 3 1

3°/29 γ 14 125 TGTGCTAG ***TG* ACTACGG *CCT*** CTACTGG 2 1

1°7/08  1 386 TGT ***GCAAGGG* CTACGG *CCATGGGGG*** CCTGGTTTGCTTACTGG 3 2

1°7/25 γ 3 128 TGTGCAAGA ***GGACTCT* TAGTT** -TACTGGTACTTCGATGTCTGG 1 2

1°14/11 γ 2 171 TGTGCCAGA ***GATTT* CGGTA *AGG*** ACTGG 3 1

2°/07^l^ γ 2 171 TGTGCCAGA ***G* ATTAC *GGAAT*** TTACTGG 3 1

3°/11  7 158 TGTGCAAGA ***GGGGCCG* GTAGT *GGAG*** CCTGGTTTGCTTACTGG 3 1

3°/25 γ 7 158 TGTGCAAGA ***GGGG*  ACGGT *CGTGGAG*** CCTGGTTTGCTTACTGG 3 1

1°7/19 γ 1 690 TGTGCAAG ***T* GTAGCTAACTACCGTAGTAATAAA**^m^ ***CGAGGGGAAA*** ACTACTGG 2 i2

1°14/07  9 155 TGTGCAAGA ***AAGGGGG* CTACCGT**^m^ ***CACTTTTAGGGT*** GTTTGCTTACTGG 3 i1

Reverse D_H_ sequence:  **GTAGCTAACTACCGTAGTAATAAAA**

**mAb without DH segment-assignable nucleotides**^n^

3°/20 γ 2 171 TGTGCCAGA ***GATGAGGGTGTGA*** ACTGG 3 nf^o^

3°/18 γ 2 175 TGTGCCAGA ***GATTGGGGGGA*** TTATTGG 3 nf

1°7/09  2 171 TGTGCCAGA ***CTGACCCAGAC*** GTTTGCTTACTGG 3 nf

3°/19^l^ γ 2 171 TGTGCCAGA ***GATGGGGGGA*** CTTATTGG 3 nf

3°/23 γ 6 114 TGTAC ***AACTAGAGGG*** GACTATTGG 2 nf

1°7/07  1 396 TGT ***GCAATACGA*** GACTACTGG 2 nf

1°7/23^l^ γ 2 171 TGTGCCAGA ***GATCCCGGG*** GCTTACTGG 3 nf

1°7/28 γ 5 139 TGTGCAAGA ***TCGCCCGGA*** GACTACTGG 2 nf

1°7/31 γ 14 125 TGTG ***TCCCGGTGG*** CCTGGTTTGCTTACTGG 3 nf

2°/05^l^ γ 2 171 TGTGCCAGA ***GACTCCGGG*** GACTACTGG 4 nf

2°/06^l^ γ 2 171 TGTGCCAGA ***GATGGGGGG*** GCTTACTGG 3 nf

3°/04  1 286 TGTGCAAGA ***AGAGATGGG*** GCTTATTGG 2 nf

3°/05  2 171 TGTGCCAGA ***GATTCGGGG*** GACTACTGG 2 nf

1°14/03  2 495 TGTGCAA ***GATGGGAG*** GCTTACTGG 3 nf

1°14/05  2 073 TGTGCAAGA ***GACGGGGG*** TGCTTACTGG 3 nf

2°/08 γ 2 171 TGTGCCAGA ***GATGGGGG*** GGACTACTGG 4 nf

1°7/27 γ 5 163 TGTGCAAGA ***CATAACA*** ACTATGCTATGGACTACTGG 4 nf

3°/10  6 494 TGTACGG ***GGGGTC*** CCTGGTTTGCTTACTGG 3 nf

1°7/04  1 627 TGTGCAAGA ***GCGA*** ACTTTGACTACTGG 2 nf

1°7/03  1 627 TGTGCAAG ***GG*** GGTACTTCGATGTCTGG 1 nf

1°7/12  3 128 TGTGCAAG ***GC*** GGTACTTCGATGTCTGG 1 nf

1°7/24 γ 3 128 TGTGCAAG ***GC*** GGTACTTCGATGTCTGG 1 nf

1°7/01  1 673 TGTGCAAGA ***G*** GGTACTTCGATGTCTGG 1 nf

Legend

^a^ The annotation of these 83 antibodies indicates their generation after primary immunization on day 7 (1°7) or day 14 (1°14), secondary (2°) and tertiary (3°) TD immunization and a specific number.

^b^ Isotype of antibodies.

^c^ V_H_ genes: F – V_H_ family, no. – gene number according to VBASE2, nucleotide sequence of 3’-end.

^d^ N nucleotides are marked in bold italic letters and, in addition, P nucleotides are underlined.

^e^ Antibodies are arranged in descending lengths of D_H_ segment-encoded nucleotides which are marked in bold. For comparison, the full D_H_ segment of ΔD-iD mice is shown in the first line underneath.

^f^ Shows the nucleotide sequence of the 5’-end of the J_H_ gene segment and its respective number.

^g^ Reading frame: RF1 – 42.17%; RF2 – 27.7%; RFi1 – 1.2%; RFi2 – 1.2%; RF n.f. – 27.7%

^h^ This mAb shows 2 separated stretches with sequence homology to the D_H_ segment of ΔD-DμFS mice.

^i^ These 2 antibodies have identical V_H_ and CDR-H3, but different V_L_.

^k^ These 2 antibodies have identical CDR-H3 and V_L_, but different V_H_.

^l^ These antibodies on gray background are encoded by the genetic Id_Ox1_ gene combination VH171/Vκ072.

^m^ Nucleotides encoded by complementary stretches of the D_H_ gene segment are surrounded by frames. The full complementary sequence of the D_H_ segment of ΔD-DμFS mice is given underneath.

^n^ Antibodies with no D_H_ segment-assignable nucleotides; N/P nucleotides are shown as a single group since a correlation to the D_H_-J_H_ or V_H_-D recombination is not possible.

^o^ nf = not found

**Supplementary Table 3b**

CDR-H3 nucleotide sequences of anti-phOx antibodies from D-altered ΔD-iD mice

**V_H_ gene**^c^

**mAb**^a^ **Is**^b^ **F** **no.** **3’-end N/P**^d^ **D_H_ segment-encoded**^e^ **N/P**^d^ **J_H_**^f^ **RF**^g^

**D_H_ of ΔD-iD mice: TTTATCGTAATCATAGTAGAAGCTAC**

3°/06  5 147 TGTGCAAGA ***ATCA*** **ATCGTAATCATAGTAGAAGCTAC *CCGGGG*** TACTTTGACTACTGG 2 1

1°7/01  1 532 TGTGCAAGA ***AGGGG* TCGTAATCATAGTAGAAGCTAC *GTAGGCG*** GGTACTTCGATGTCTGG 1 1

3°/07  5 135 TGTGCAAG ***TA*  TTTATCGTAATCATAGTAGAAG *TC*** ACTGGTACTTCGATGTCTGG 1 1

1°7/07  5 131 TGTGCAAGA ***CAGGGG* CGTAATCATAGTAGAAGCTAC *CC*** CTACTGGTACTTCGATGTCTGG 1 1

1°14/03  1 014 TGT ***GCAAGACGAG* ATCGTAATCATAGTAGAAGCT *TCCT*** CTACTGGTACTTCGATGTCTGG 1 1

1°14/07 5 185TGTGCAAG ***GTCAA*** **TCGTAATCATAGTAGAAGCTA *AACTA*** TGG 2 2

1°14/11 5 135TGTGCAAGA ***CCAGGA*** **CGTAATCATAGTAGAAGCTAC** ***CC*** CTACTTTGACTACTGG 2 1

1°7/13 γ 2 171 TGTGCCAG ***GTC* TTATCGTAATCATAGTAGAA *CC*** GCTTACTGG 3 1

1°14/04  3 128 TGTGCAAG ***GGG* CGTAATCATACTACTAGCTA *GA*** TACTTTGACTACTGG 2 2

1°14/05 3 128TGTGCAAGA ***AAGG* ATCGTAATCATAGTAGAAG *GG*** ACTGGTACTTCGATGTCTGG 1 1

1°14/10 5 135TGTGCAA - **ATCGTAATCATAGTAGAAG** - CTACTGGTACTTCGATGTCTGG 1 1

3°/01  2 201 TGTGCCAAA ***ATGGG* TCGTAATCATAGTAGAAG** - CTACTTTGACTACTGG 2 1

1°14/12 5 135TGTGCAAGA ***CATCGG* CGTAATCATAGTAGAAG *AGAGACTG*** ACTGGTACTTCGATGTCTGG 1 1

1°14/06 5 192TGTGCAAGA ***CCTT* TTTATCGTAATCATAG *GGGA*** TACTTTGACTACTGG 2 1

1°14/02 1 286TGTGCAAGA ***C*** **ATCATAGTAGAAG** - CTACTGGTACTTCGATGTCTGG 1 1

3°/04  2 171 TGTGCCAGA ***GCGG* GTAGAAGCTAC *GGG*** TGGTACTTCGATGTCTGG 1 1

1°7/16 γ 6 114 TGTACCAGG ***CCTC* TTATCGTAA *GGGTTCAC*** TTTGCTTACTGG 3 2

1°14/14  19 028 TGTGTGAG ***ACATGGGA* CGTACTCAT *GGAGA*** GACTACTGG 2 1

1°14/19γ1 455TGTACAAGA ***TCATGGGGGG* ATCATAGTA *AAACCTACGGTT*** CCTGGTTTGCTTACTGG 3 1

2°/02 γ 1 386 TGTGCAAGA ***CTA* CGTAATCAT *GGA*** GACTCTTGG 2 1

1°7/15 γ 5 139 TGTGCAAGA ***CC* CGTAATCA *GATA*** TTACTATGCTATGGACTACTGG 4 2

1°7/02  1 532 TGTGCAAG ***GA* TTTATC *TGATCC*** CTTACTGG 3 1

1°14/09  5 147 TGTGCAAGA ***CACCC* GCTAC *GACG*** CTACTTTGACTACTGG 2 3

1°7/14 γ 5 176 TGTGCAA **GTAGCTTCTACTATGATTACGATAAA**^h^  ***TC*** GTTTGCTTACTGG 3 i2

1°14/08  5 178 TGTACAAGA **GTAGCTTCTACTATGATTACGGTAAA *GAGGGTCC*** TTGACTACTGG 2 i1

3°/05  5 192 TGTGCAAGA ***TCCCG* TTACGAT** -TACTATGCTATGGACTACTGG 4 i2

3°/12 γ 3 120 TGTGTAAGA ***GGAGGGACGGTA* GTAGCT** -TTTGACTACTGG 2 i1

3°/13 γ 7 158 TGTGCAAGA ***GGGTTG* TACGA *GGGAG*** CCTGGTTTTCTTATTGG 3 i2

Reverse D_H_ sequence: **GTAGCTTCTACTATGATTACGATAAA**

**mAb without D_H_ segment-assignable nucleotides**^i^

1°14/13  6 114 TGTACCAGG ***CCCGTCATCCCAGAGG*** CCTGGTTTGCTTACTGG 3 nf^k^

3°/09^l^ γ 1 386 TGTGTAAGA ***AAGTTCCGAGGGGG*** TGACTACTGG 2 nf

3°/10^l^ γ 1 386 TGTGTAAGA ***AAGTTCCGAGGGGG*** TGACTACTGG 2 nf

3°/03^m^  2 171 TGTGCCAGA ***GATGGAGGGAT*** TTCCTGG 3 nf

1°7/03  1 286 TGTGCAAGA ***TGGGGCAAC*** GACTACTGG 2 nf

1°7/04  2 171 TGTGCCAGA ***GATCGGGGG*** GACTACTGG 2 nf

1°7/05  2 171 TGTTCCAGA ***GATCGGGGG*** GACTACTGG 2 nf

1°7/06  2 171 TGTGCCAGA ***GATCGGGGG*** GCTTACTGG 3 nf

3°/02  2 171 TGTGCCAGA ***GATGGGGGG*** GACTACTGG 2 nf

1°7/11 γ 2 183 TGTGCCAGA ***AATTGGGG*** GGACTACTGG 4 nf

1°7/12 γ 2 171 TGTGCCAGA ***GATCGAGG*** GGACTACTGG 4 nf

1°14/01  1 706 TGTGCAAGA ***AGAGATGC*** CTACTGG 2 nf

3°/11^m^ γ 2 171 TGTGCCAGA ***GATGGAGG*** TGCTTTCTGG 3 nf

1°7/08  6 114 TGTACCAGG ***CGAGGGA*** CTCACTGG 3 nf

2°/01  6 114 TGTACCAGG ***CGGGGC*** GACTACTGG 2 nf

1°14/18 γ1 528TGTGCAAGA ***CGC*** TTTGCTTACTGG 3 nf

Legend

^a^ The annotation of these 44 antibodies indicates their generation after primary immunization on day 7 (1°7) or day 14 (1°14), secondary (2°) and tertiary (3°) TD immunization and a specific number.

^b^ Isotype of antibodies.

^c^ V_H_ genes: F – V_H_ family, no. – gene number according to VBASE2, nucleotide sequence of 3’-end.

^d^ N nucleotides are marked in bold italic letters and, in addition, P nucleotides are underlined.

^e^ Antibodies are arranged in descending length of D_H_ segment-encoded nucleotides which are marked in bold. For comparison, the full D_H_ segment of ΔD-iD mice is shown in the first line underneath.

^f^ Shows the nucleotide sequence of the 5’-end of the J_H_ gene segment and its respective number.

^g^ Reading frame: RF1 – 40.9%; RF2 – 9.1%; RF3 – 2.27%; RFi1 – 4.54%; RFi2 – 6.81%; RF n.f. – 36.36%

^h^ Nucleotides encoded by complementary stretches of the D_H_ gene segment are surrounded by frames. The full complementary sequences of the D_H_ segment of ΔD-iD mice are given underneath.

^i^ Antibodies with no D_H_ segment-assignable nucleotides; N/P nucleotides are shown as a single group since a correlation to the D_H_-J_H_ or V_H_-D_H_ recombination is not possible.

^k^ nf = not found

^l^ These 2 antibodies have identical VH, CDR-H3 and V_L_, but slightly different CDR-L3.

^m^ These antibodies on gray background are encoded by the genetic Id_Ox1_ gene combination VH171/VK072.

**Supplementary materials References**

1. Lange H, Zemlin M, Tanasa RI, Trad A, Weiss T, Menning H, et al. Thymus-independent type 2 antigen induces a long-term IgG-related network memory. Mol Immunol. 2008;45(10):2847-60. doi: 10.1016/j.molimm.2008.01.020

2. Lange H, Hecht O, Zemlin M, Trad A, Tanasa RI, Schroeder HW, Jr., et al. Immunoglobulin class switching appears to be regulated by B-cell antigen receptor-specific T-cell action. Eur J Immunol. 2012;42(4):1016-29. doi: 10.1002/eji.201141857

3. Eisenberg D. Three-dimensional structure of membrane and surface proteins. Annu Rev Biochem. 1984;53:595-623.
